# Spatial positioning of preimplantation mouse embryo cells is regulated by mTORC1 and m^7^G-cap-dependent translation at the 8- to 16-cell transition

**DOI:** 10.1098/rsob.230081

**Published:** 2023-08-09

**Authors:** Lenka Gahurova, Jana Tomankova, Pavlina Cerna, Pablo Bora, Michaela Kubickova, Giorgio Virnicchi, Kristina Kovacovicova, David Potesil, Pavel Hruska, Zbynek Zdrahal, Martin Anger, Andrej Susor, Alexander W. Bruce

**Affiliations:** ^1^ Laboratory of Early Mammalian Developmental Biology (LEMDB), Department of Molecular Biology and Genetics, Faculty of Science, University of South Bohemia, Branišovská 31, 37005 České Budějovice, Czech Republic; ^2^ Laboratory of Biochemistry and Molecular Biology of Germ Cells, Institute of Animal Physiology and Genetics, Czech Academy of Sciences, Rumburská 89, 27721 Liběchov, Czech Republic; ^3^ Laboratory of Cell Division Control, Institute of Animal Physiology and Genetics, Czech Academy of Sciences, Rumburská 89, 27721 Liběchov, Czech Republic; ^4^ Department of Genetics and Reproduction, Central European Institute of Technology, Veterinary Research Institute, Hudcova 296/70, 621 00 Brno, Czech Republic; ^5^ Laboratory of Functional Genomics and Proteomics, National Centre for Biomolecular Research, Faculty of Science, Masaryk University, Kamenice 753/5, 62500 Brno, Czech Republic; ^6^ Central European Institute of Technology, Masaryk University, Kamenice 753/5, 62500 Brno, Czech Republic

**Keywords:** mTOR/mTORC1, EIF4EBP1/4EBP1, TOP-motif, preimplantation mouse embryo, cell fate, inner cell mass/ICM and cell positioning

## Abstract

Preimplantation mouse embryo development involves temporal–spatial specification and segregation of three blastocyst cell lineages: trophectoderm, primitive endoderm and epiblast. Spatial separation of the outer-trophectoderm lineage from the two other inner-cell-mass (ICM) lineages starts with the 8- to 16-cell transition and concludes at the 32-cell stages. Accordingly, the ICM is derived from primary and secondary contributed cells; with debated relative EPI versus PrE potencies. We report generation of primary but not secondary ICM populations is highly dependent on temporal activation of mammalian target of Rapamycin (mTOR) during 8-cell stage M-phase entry, mediated via regulation of the 7-methylguanosine-cap (m^7^G-cap)-binding initiation complex (EIF4F) and linked to translation of mRNAs containing 5′ UTR terminal oligopyrimidine (TOP-) sequence motifs, as knockdown of identified TOP-like motif transcripts impairs generation of primary ICM founders. However, mTOR inhibition-induced ICM cell number deficits in early blastocysts can be compensated by the late blastocyst stage, after inhibitor withdrawal; compensation likely initiated at the 32-cell stage when supernumerary outer cells exhibit molecular characteristics of inner cells. These data identify a novel mechanism specifically governing initial spatial segregation of mouse embryo blastomeres, that is distinct from those directing subsequent inner cell formation, contributing to germane segregation of late blastocyst lineages.

## Graphical abstract

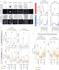

## 1. Introduction

Preimplantation stages of mouse embryo development culminate at E4.5 with formation of peri-implantation blastocysts, comprising three distinct cell lineages. These are two differentiating and epithelized lineages, the outer trophectoderm (TE—ultimately contributing to placenta) and the primitive endoderm (PrE—a monolayer residing at the cavity to inner-cell-mass (ICM) interface, later forming yolk sac membranes). The third lineage is the pluripotent epiblast (EPI—deep within the ICM, serving as a foetal cell progenitor pool) [1,2]. Cleavage of apical-basolaterally polarized 8-cell stage blastomeres [3–7] heralds the first relative spatial segregation of embryonic cells. Resultant daughter 16-cell stage blastomeres either occupy outer positions with contactless apical domains and intact polarity (and undergo TE differentiation) or are positioned on the inside as surrounded apolar cells that remain pluripotent [8–11]. Following the 16- to 32-cell transition, a secondary group of apolar inner cells is similarly supplemented, from outer polarized parental cells, to the primary inner cell progeny population, that together constitute a nascent early blastocyst (E3.5) ICM from which EPI and PrE are derived [12]. Traditionally, relative spatial positioning of blastocyst lineage progenitors was considered via a prism of cell division plane orientation; characterized as those aligning along the embryonic radial axis (i.e. apical-basolateral polarity axis) generating an apolar inner and a polarized outer cell (termed ‘asymmetric/differentiative’ divisions) and those occurring perpendicularly yielding two polarized outer cells (via ‘symmetric/conservative’ cleavages [13]). More recent time-lapse studies indicate most divisions broadly correlate with mitotic spindle alignments along the embryonic radial axis but only a fraction of apolar inner cells are directly deposited post-cytokinesis [14–17]. Indeed, most blastomeres adopt relative spatial positions after oblique-angled divisions, typically resulting in two initially outer residing daughters with significantly unequally sized contactless apical domains. In such situations, increased actomyosin driven cortical tension causes internalization of cells with smaller contactless apical domains [15,16]. A role for intra-cellular apical-basolateral polarity in regulating cell internalization is supported by clonal dysregulation of the apical polarity factor PRKCZ/I resulting in blastomere internalization [18] and the spontaneous internalization of naturally occurring apolar outer 16-cell stage blastomeres [15]; also reviewed in [19,20]. Whether relative spatial positioning, and consequent blastocyst cell fate, is essentially a stochastic process subject to intrinsic mitotic spindle angle and cell division plane orientation heterogeneity [21,22] or subject to other contributing factors (e.g. cell shape, intercellular contact or intrinsic organization of individual cells [23]) remains unclear. For example, apical to basal repositioning of 8-cell stage nuclei is reported to positively correlate with increased incidence of inner cell generation [24] and positive correlations between polarized contactless apical domain size and mitotic spindle angle orientation along the radial embryonic axis are described [21] supported by studies on isolated 8-cell blastomeres, that are nevertheless regulated in relative frequency in intact dividing 8-cell stage embryos [25].

Spatial separation of polarized outer TE progenitors from nascent apolar ICM blastocyst populations defines the ‘first cell fate decision’ [1,2] and is accompanied by distinct lineage marker gene expression. Outer TE progenitors express the transcription factor CDX2 [26,27] and early blastocyst ICM cells co-express pluripotency-related transcriptional regulators NANOG [28,29] and SOX2 [11,30,31] with the early PrE marker GATA6 [32,33] indicative of apparent uncommitted ICM fate preceding the ‘second cell fate decision’. These expression domains are regulated via differential activation of Hippo-signalling, ultimately supressed in a polarity-dependent mechanism in emerging outer TE cells. This is achieved via apical domain sequestration of the Hippo activator AMOT [9,34] and activated in apolar ICM founders to resist TE differentiation and promote pluripotency [10,11,35]. However, early blastocyst ICM is comprised of both primary and secondary ICM founders, each exposed to varying degrees of past Hippo-pathway activation and suppression, thus questioning their relative capacities to preferentially derive EPI or PrE. A debated model contends primary ICM founders, subjected to relatively earlier Hippo-signalling activation, preferentially contribute EPI and secondary inner cells, initially sequestered from active Hippo-signalling and exposed to additional TE-differentiative cues as outer 16-cell stage blastomeres, are strongly biased towards PrE [14,36–43]. Hence, enhanced mechanistic insight into relative spatial segregation of polarized outer TE-progenitors from both primary and secondary ICM founders will assist understanding of the potentially integrated nature of blastocyst lineage derivation.

The mammalian target of Rapamycin (mTOR) is an evolutionarily conserved serine/threonine kinase of the phosphoinositide 3-kinase family, serving as the central metabolic cellular regulator in response to various intrinsic and extrinsic stimuli. mTOR integrates upstream signalling inputs with downstream effectors, including components of the transcriptional and translational apparatus, to functionally regulate key processes of energy utilization, specific metabolic pathways, cell growth and proliferation, autophagy and protein synthesis and degradation. mTOR is the catalytic subunit of two distinct complexes, mTORC1 and mTORC2, respectively, regulating cell growth (e.g. lipid and nucleotide synthesis, protein synthesis and degradation and autophagy) and survival/proliferation (e.g. apoptosis, glucose metabolism, ion transport and cytoskeleton rearrangement). mTOR activity within mTORC1 but not mTORC2 can be inhibited by the compound Rapamycin, whereas second generation ATP analogue inhibitors, such as Torin1, are effective against both complexes [44–47]. Active mTORC1 regulates protein translation via phosphorylation of many translation initiation factors and ribosomal related proteins, including the key effectors, eukaryotic translation initiation factor 4E-binding proteins (EIF4EBP1/2/3 [48]). Unphosphorylated EIF4EBPs inhibit 7-methylguanosine-cap (m^7^G-cap)-dependent translation by direct sequestration of the m^7^G-cap-binding-complex protein EIF4E from the EIF4F translation initiation complex (also comprising the scaffold protein EIF4G1 and RNA helicase EIF4A [49,50]). Hence, direct mTOR mediated EIF4EBP phosphorylation impairs this inhibitory interaction and facilitates EIF4F initiation complex-driven m^7^G-cap-dependent translation (reviewed in [45,47]. However, sensitivity of specific mRNA translation to mTOR/mTORC1 inhibition (mTORi) is not uniform. Transcripts containing so-called TOP- (5′-UTR terminal oligopyrimidine) or TOP-like sequence motifs (collectively referenced here as TOP-motifs), often but not exclusively derived from genes related to protein synthesis itself, are significantly more sensitive to mTORi. Therefore, TOP-motif presence, most conservatively defined as a m^7^G-capped C nucleotide followed by a run of 4–15 pyrimidines [51], identifies mRNA transcript classes that are selectively transcribed under conditions of enhanced active mTOR signalling, predominantly via phosphorylation of EIF4EBP [49]. Additionally, the mTORC1 substrate and RNA-binding protein LARP1, regulates TOP-motif mRNA translation by directly interacting, in its unphosphorylated state, with TOP-motifs to inhibit translation (an association impaired upon mTORC1 dependent phosphorylation), although this model is disputed (reviewed in [45,52]).

In the field of mammalian reproduction and early development, mTOR was mechanistically studied during mouse oocyte meiotic development. Pharmacological inhibition of spindle associated mTOR impairs cortical spindle migration, asymmetric extrusion of the first meiotic polar body and causes cytoskeletal disruption [53]. Moreover, mTOR-mediated inactivation of EIF4EBP1 (via phosphorylation of specific substrate residues) facilitates appropriate spatio-temporal translation of germinal vesicle enriched or spindle proximal mRNAs important for meiotic maturation, including those with TOP-motifs [54] (e.g. encoding ANK2 [55]). Post-fertilization, mTOR directed phosphorylation of EIF4EBP1 is reported as an important translational regulator of the maternal-to-embryonic transition [56]. In early mouse blastocysts (but not earlier cleavage stages), partial pharmacological mTORi (targeting mTORC1 and mTORC2) results in prolonged but reversible and viable *ex vivo* paused development, akin to natural hormonally induced *in vivo* diapause [57]. Additionally, mTOR signalling, operating downstream of active p38-MAPK, is distinctly implicated during PrE (but not EPI) specification from uncommitted early mouse blastocyst ICM populations, whereby defective PrE specification caused by p38-MAPK inhibition [58,59] is associated with reduced protein translation [60]. Such reports exemplify mTOR associated and lineage-specific mechanisms regulating early developmental cell fate, further supported by observations of relative differences in mTOR activity underpinning necessary cellular competition and elimination in early post-implantation embryonic tissues exiting naïve pluripotency [61].

Here, we report enhanced levels of M-phase associated phospho-EIF4EBP1 (pEIF4EBP1) around the 8- to 16-cell transition, sensitive to mTORi. mTORi around this transition results in the generation of fewer 16-cell stage primary ICM founder cells without affecting apical-basolateral polarity in supernumerary outer cells. Dysregulation of EIF4EBP1, LARP1 and EIF4F m^7^G-cap-binding-complex function phenocopies mTORi, as does siRNA mediated clonal knockdown of identified TOP-motif containing mRNAs related to the cytoskeleton and secondary RNA structure. These data invoke a mechanism by which mTORC1 activity at the 8- to 16-cell transition facilitates translation of specific TOP-motif containing mRNAs, functionally required to generate primary ICM founder cells, reported to preferentially contribute EPI [14,38,40]. However, we also report a lack of a similar active mTOR requirement to generate secondary ICM founders around the 16- to 32-cell transition, suggesting distinct mechanisms of lineage relevant blastomere spatial segregation after successive cleavage rounds. While early blastocysts (E3.5) cultured from the 8-cell stage under mTORi conditions present with fewer ICM cells, resulting late (E4.5) blastocysts (derived when such embryos are transferred to regular culture conditions) recover ICM cell number, specify an appropriately sized EPI but present with evidence of impaired PrE differentiation.

## 2. Results

### 2.1. mTORC1 signalling during 8-cell stage M-phase entry is associated with generation of primary ICM founders at the 16-cell stage and mTOR-EIF4EBP1-EIF4E/mRNA cap-binding-complex axis function

**Figure 1.**
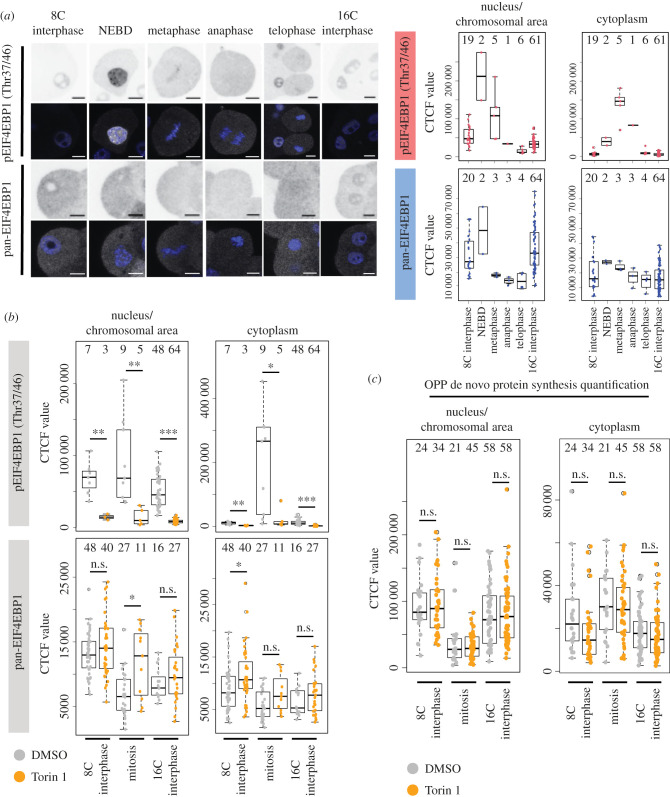
Enhanced mTOR-dependent expression levels of pEIF4EBP1 during the 8- to 16-cell cleavage division. (*a*) Example IF staining micrographs of pEIF4EBP1 (Thr37/46) and pan-EIF4EBP1 at either 8- or 16-cell interphase and the individual stages of mitotic division from 8- to 16-cell stage in individual blastomeres (left), as quantified separately for nucleus (interphase cells)/chromosomal area defined by DAPI staining (mitotic cells) and cytoplasm (right). EIF4EBP1 visualized in greyscale, DAPI in blue, representative confocal *z-*stacks for individual stages shown. Scale: 20 µm. (*b*) Quantification of IF staining of pEIF4EBP1 (Thr37/46) and pan-EIF4EBP1 in 8- and 16-cell interphase and dividing blastomeres, +/− Torin1. (*c*) Quantification of nascent translation by O-propargyl-puromycin (OPP) polypeptide incorporation and fluorescent labelling assay in 8- and 16-cell interphase and dividing blastomeres, +/− Torin1. In all graphs, numbers of analysed blastomeres are shown.

Motivated by published meiotic phenotypes associated with mTORi during oocyte maturation and defective asymmetric polar body extrusion [53–55,62,63], we assayed potential functional mTOR roles during the first spatial cellular separation in preimplantation mouse embryos. We assayed levels of phospho-4EIF4EBP1 (p4EIF4EBP1), a known product of active mTORC1 signalling [64], at the 8- to 16-cell transition and noted increased p4EIF4EBP1 levels associated with 8-cell stage M-phase entry, that were localized around condensing chromosomes and associated with mitotic spindles, returning to basal levels in 16-cell stage progeny (figure 1*a*). Torin1 mediated mTORi showed reduction in pEIF4EBP1 levels across all tested developmental timepoints. By contrast, overall EIF4EBP1 expression did not change between control and mTORi treated groups at 8- and 16-cell interphase, and it was even increased after mTORi during M-phase (figure 1*b*; electronic supplementary material, figure S1a). We interpret this as indicating an M-phase specific increase in mTOR signalling during the 8- to 16-cell transition. We next determined if this increase was associated with elevated general de novo translation, using an O-propargyl-puromycin (OPP) polypeptide incorporation and fluorescent labelling assay. Despite the observed increase in mTORi sensitive pEIF4EBP1 levels, we did not detect a significant difference in de novo translation during this period (figure 1*c*; electronic supplementary material, figure S1b), suggesting basal protein synthesis was not affected. We hypothesized observed increases in pEIF4EBP1 levels may not affect global mRNA translation but rather a small subset of functionally significant transcripts, such as those described in oocytes harbouring TOP-motifs [55].

**Figure 2.**
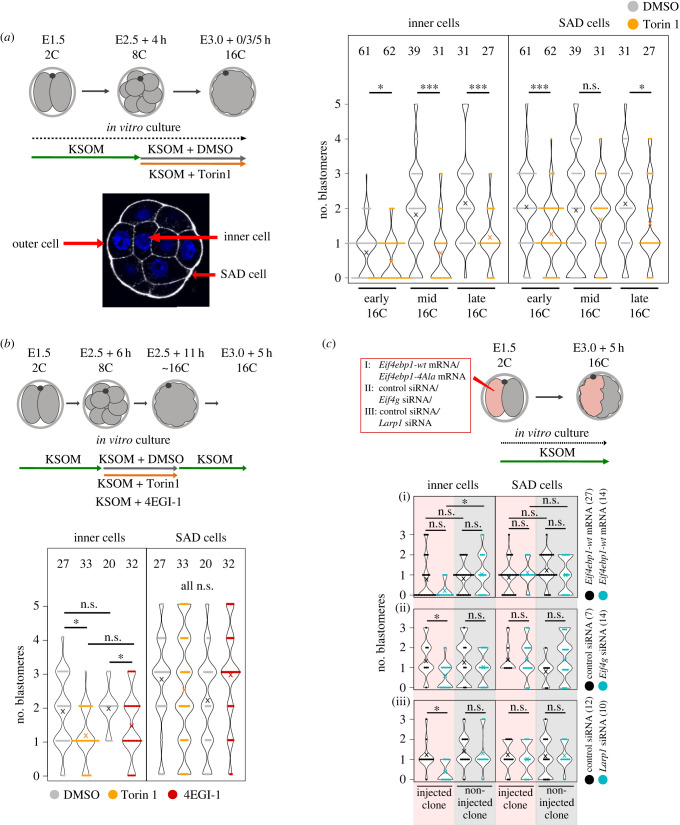
mTOR-regulated m^7^G-cap-dependent translation plays a role in primary ICM cell generation. (*a*) Experimental scheme, visualization of example outer, inner and SAD cells, and quantification of the number of inner and SAD cells in 16-cell embryos, +/− Torin1. (*b*) Experimental scheme with shorter inhibition period and quantification of inner and SAD cells in 16-cell embryos, +/− Torin1 or +/− 4EGI-1. (*c*) Experimental scheme and quantification of inner and SAD cells in injected (i.e. indicated siRNA/mRNA) and non-injected clones of 16-cell stage embryos. In all graphs, ‘×’ marks the average value, and numbers of analysed embryos are shown.

We noted Torin1 mediated mTORi from the mid-8-cell stage also resulted in significantly fewer inner cells in early-, mid- and late-16-cell stage morulae, with multiple examples of embryos lacking any primary ICM founder population, when compared with DMSO treated controls. We also noted equal numbers (mid-16-cell) and decreased (early- and late-16-cell) numbers of blastomeres that maintained a minimal outer contact, that we termed small apical domain (SAD) cells (figure 2*a*; electronic supplementary material, table S1). This suggests that lower numbers of inner cells in mTORi are not compensated by increased numbers of SAD cells that will be later internalized. Furthermore, we observed a similar mTORi phenotype using the alternative inhibitor Rapamycin, suggesting the phenotype is mediated via impaired mTORC1 function [44–47] (electronic supplementary material, figure S2a). Shortening the mTORi exposures (using Torin1) revealed the phenotype of deficient primary ICM founders was minimally centred during a 5 h window around 8-cell stage mitotic onset and entry into the 16-cell stage (figure 2*b*; electronic supplementary material, table S2). Inhibition of the formation of the EIF4F initiation complex using the compound 4EGI1 (that blocks association of the EIF4F mRNA m^7^G-cap-binding-complex subunits, EIF4E and EIF4G [65]) during the same 5 h window generated a mTORi phenocopy of fewer primary ICM founder cells, confirming the phenotype results from functional dysregulation of the mTOR-EIF4EBP1-EIF4E/mRNA cap-binding-complex axis (figure 2*b*; electronic supplementary material, table S2). To further confirm this conclusion, we employed a known dominantly acting recombinant 4EIF4EBP1 construct (in which four known mTOR specific phosphorylation amino acid substrates are mutagenized to alanine; i.e. 4Ala-EIF4EBP1 [49]), as its expression should impair elevated levels of m^7^G-cap-dependent mRNA translation, irrespective of mTOR signalling status. Accordingly, *in vitro* transcribed mRNA encoding 4Ala-EIF4EBP1 (or wild-type EIF4EBP1—incorporating a N-terminal HA-epitope tag) was microinjected (plus a lineage injection marker; mRNA encoding Histone-H2B-YFP) into one blastomere of 2-cell stage embryos that were then cultured until the mid-16-cell stage. The expression of the recombinant 4Ala-EIF4EBP1 protein was confirmed by immuno-fluorescent (IF) staining (electronic supplementary material, figure S2b—at the biologically relevant, regarding mTORi, 8- to 16-cell transition). As after mTORi, we observed reduced primary ICM founder cell contribution, restricted to the marked progeny of the microinjected clone, although microinjection of a similar wild-type EIF4EBP1 recombinant mRNA had no significant effect (figure 2*c*; electronic supplementary material, table S3). We interpreted these data as further evidence of the involvement of the mTOR-EIF4EBP1-EIF4E/mRNA cap-binding-complex axis as a component of the observed mTORi phenotype. We used a similar microinjection strategy to downregulate the mRNAs encoding EIF4G1 (one of three subunits of the EIF4F m^7^G-cap-binding-complex [64]) and *Larp1* (a mTOR substrate implicated in efficient mRNA translation, particularly of TOP-motif containing mRNAs [45,52]) using specific siRNAs (after first confirming efficient siRNA mediated target transcript knockdown by quantitative RT-PCR of siRNA microinjected in 2-cell stage embryos, targeting both blastomeres, cultured to the mid-16-cell stage—electronic supplementary material, figure S2c). We again elicited phenocopies previously associated with mTORi involving reduced and clonal primary ICM founder cell contribution (figure 2*c*; electronic supplementary material, figure S2d and table S3). Collectively, these data indicate an 8-cell stage and M-phase specific temporal boost in mTORC1 activity, acting via the mTOR-EIF4EBP1-EIF4E/mRNA cap-binding-complex axis, potentiates deposition of daughter blastomeres to the inner compartment of 16-cell stage embryos as primary ICM founding cells.

### 2.2. mTORi does not affect apical-basolateral polarization, relative nuclear positioning upon 8-cell stage M-phase entry nor mitotic spindle orientation

Relative spatial positioning of outer and inner cells from the 16- until the 32-cell stage is known to be tightly regulated by apical-basolateral polarity (established at the late 8-cell stage [13]); whereby in outer blastomeres lacking apical-basolateral polarity, increased relative actomyosin contractility actively segregates cells to the inner compartment [15,16,18,19]. We therefore assayed the expression of polarity related proteins in 16-cell stage embryos exposed to mTORi from the late 8-cell stage using IF, assaying the apical polarity factor aPKC/PRKCZ [18], basolaterally localized cell adhesion protein ECAD/CDH1 [66] and YAP1 (as a readout of polarity-dependent Hippo-pathway activity). We did not observe any difference in apical polarity status of embryos with supernumerary outer cells after mTORi and any generated inner cells were appropriately apolar (electronic supplementary material, figure S3a). Similarly, ECAD expression was appropriately basolateral in outer cells, although there was a small and significant reduction in the quantified levels of protein expression at such membranes (electronic supplementary material, figure S3b). Compared to controls, the relative numbers of blastomeres exhibiting nuclear or cytoplasmic YAP1 expression remained unchanged, indicating correct polarity-dependent regulation of the Hippo-pathway (electronic supplementary material, figures S3c, S3d). These data indicate the mTORi phenotype of fewer primary ICM founders is not related to regulation of apical-basolateral polarity.

**Figure 3.**
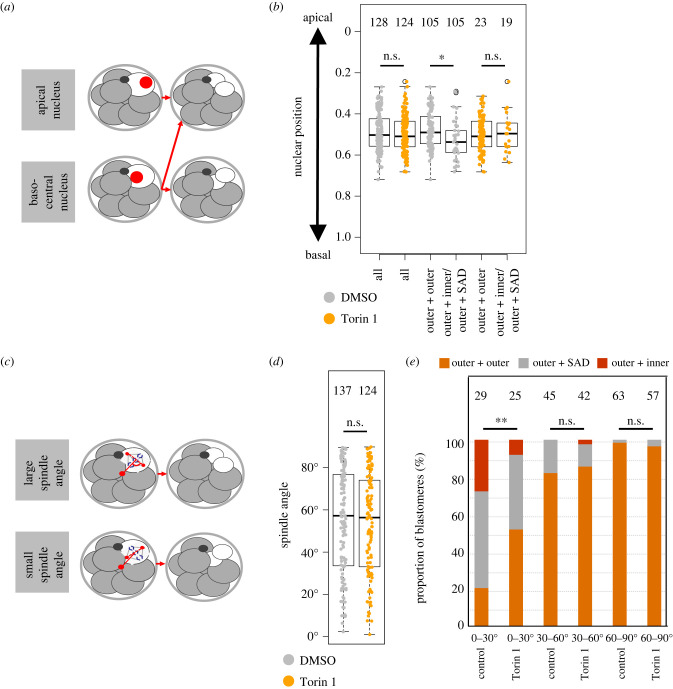
Trends associated with 8-cell stage blastomere nuclear positioning and mitotic spindle angles and the generation of primary ICM cells are not applicable under mTORi conditions. (*a*) Scheme of the identified association between 8-cell stage blastomere nuclear position and spatial positions of daughter cells post-division [24]. (*b*) Quantification of the position of the immediately pre-M-phase 8-cell stage nucleus on the intra-cellular apico-basolateral axis (i.e. embryonic radial axis), +/− Torin1; in all blastomeres, or those that generated two outer cells or one outer and one inner/SAD daughter cell at the 16-cell stage. Numbers of analysed blastomeres are shown. (*c*) Scheme of the reported association between mitotic spindle angle and spatial positions of daughter blastomeres after 8-cell stage cell division; note: spindle angle is denoted by the angle of lines bisecting the two spindle poles and the radial axis of the embryo from each individual blastomere’s most apical membrane domain. (*d*) Quantification of the average spindle angle of 8-cell dividing blastomeres, +/− Torin1. Numbers of analysed blastomeres are shown. (*e*) Quantification of the spindle angle versus resulting spatial positions of daughter blastomeres, +/− Torin1. Each bar represents at least 15 blastomeres. Numbers of analysed blastomeres are shown.

We next employed live fluorescent confocal microscopy embryo imaging to observe individual 8-cell blastomere division. Recovered 2-cell stage embryos were microinjected in both blastomeres with three recombinant mRNAs encoding differentially labelled fluorescent reporter protein constructs (i.e. Histone-H2B-mCherry, GAP43-CFP and alpha-Tubulin-Venus, to visualize chromatin, plasma membranes and tubulin-cytoskeleton/mitotic spindle, respectively [60,67]). Microinjected embryos were cultured until the late 8-cell stage and exposed to mTORi (using Torin1) or solvent control DMSO before imaging through the 8- to 16-cell stage transition. We measured relative 8-cell stage nuclei positioning along the embryonic radial axis (determined from the most apical component of each blastomere) upon M-phase entry and the spatial fate of daughter cells by the late 16-cell stage. In control embryos, we observed previously reported trends describing increased incidence of inner cell generation associated with more basal nuclear positioning and the generation of two outer cells being linked with apical positioning [24] (figure 3*a*). In mTORi treated groups, we did not observe any difference in the overall frequency nor distribution of relative nuclear positioning observed in control embryos. However, we found the confirmed trends relating to 16-cell stage blastomere positioning no longer held. Indeed, the observed bias for basal nuclear position yielding an outer and inner/SAD daughter blastomere in the control group is not present in mTORi treated embryos (figure 3*b*). These results indicate previously published outcomes associated with nuclear positioning are nevertheless sensitive to mTORi, even if the frequency and distribution of nuclear positioning were not affected. We next measured relative mitotic spindle angles in relation to the radial axis of each 8-cell stage blastomere (by intersecting a line drawn through the opposing spindle poles and the defined embryonic radial axis; figure 3*c*). Average distributions of relative spindle angles were the same between control and mTORi embryos (figure 3*d*). Nevertheless, there was a significant difference in the outcome of cell division arising from acute spindle angles (0–30°). In control conditions, these spindle angles mostly generate a single outer cell and either a SAD or inner cell, with only approximately 20% of divisions giving rise to two outer daughter cells. By contrast, in mTORi treated embryos, greater than 50% of such divisions generate two outer cells (figure 3*e*). Similarly, we interpret the data to indicate reported mechanisms related to 8-cell stage spindle angle and the propensity for primary ICM founder cell generation [24] are not operative under mTORi conditions, despite not affecting the distribution of observed spindle angles themselves. Moreover, this results in the observed supernumerary populations of outer cells and a deficit of inner cells and strongly implicates a direct role for mTORC1 signalling in post-division positioning of 16-cell stage blastomeres.

### 2.3. Dysregulation of candidate TOP-motif containing mRNA generates fewer primary ICM founders

**Figure 4.**
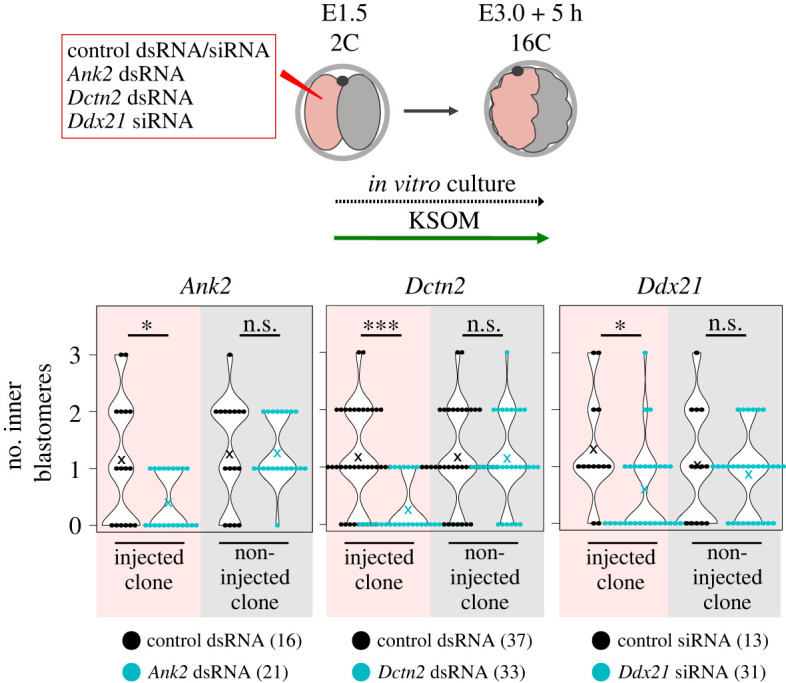
RNAi-mediated knockdown of candidate TOP-motif containing mRNAs also impairs the generation of primary ICM cells. Experimental scheme and quantification of inner and SAD cell numbers in gene/transcript specific RNAi (*Ank2*, *Dctn2* and *Ddx21*) injected and non-injected clones in 16-cell stage embryos. In all graphs, ‘×’ marks an average value. Numbers of analysed embryos are shown.

Due to the aforementioned observations (figures 1 and 2), we hypothesized that mTOR/mTORC1 might function by regulating translation of TOP-motif containing mRNAs [49,51,54]. We identified two TOP-motif containing mRNAs encoding the cytoskeletal proteins: (i) Ankyrin-2/ANK2 and (ii) Dynactin-2/DCTN2. ANK2 is a protein involved in linking integral membrane proteins to underlying spectrin-actin cytoskeleton [68] that interacts with Dynactin to promote long-range motility of cells [69] and is reported be under regulated translation by mTOR during mouse oocyte meiotic maturation [55]. DCTN2 is a component of the Dynactin macromolecular complex, an interactor of microtubules with reported roles in nuclear positioning and mitotic spindle formation [70] and is also present in a published database of candidate TOP-motif containing mRNAs [49]. After first confirming efficient dsRNA mediated knockdown of the target transcripts at the mid-16-cell stage (electronic supplementary material, figure S4a—note that both blastomeres of 2-cell embryos were microinjected with dsRNA and target mRNA levels measured by quantitative RT-PCR), we microinjected one blastomere of 2-cell stage embryos with either *Ank2* or *Dctn2* specific dsRNAs or control dsRNA (targeting GFP), plus an injection marker, and cultured them until the mid-16-cell stage. Morulae were assayed for the contribution of marked clones to primary ICM founder populations. Relating to control GFP dsRNA microinjected embryos, no significant differences of the marked or unmarked clonal contribution between outer and inner cell populations were observed. Moreover, in *Ank2* and *Dctn2* dsRNA microinjected embryos the non-injected clone did not allocate with any significant difference to equivalent clones in the GFP dsRNA control group. However, primary ICM contribution of the marked *Ank2* and *Dctn2* dsRNA microinjected clone was significantly reduced (figure 4; electronic supplementary material, figure S4b and table S4). The fact the non-microinjected clones did not exhibit increased contribution to the primary ICM population indicates a lack of any detectable enhanced compensation by this stage. We next employed an empirical mass spectrometry method to identify further candidate mRNA transcripts, involved in the generation of primary ICM founder cells, by surveying and comparing total detectable proteomes of embryos transiting the 8- to 16-cell stages under control or mTORi conditions (electronic supplementary material, table S5). Due to the scarce amount of sample material, complete proteome coverage was not obtained, but we observed a statistical depletion of DDX21 protein levels (a DEAD box RNA helicase coordinating rRNA transcription/processing during ribosome assembly [71] and a member of a family of helicases implicated in mRNA secondary structure resolution during translation [72] encoded by an mRNA containing a canonical TOP-motif [49]). Adopting a similar clonal siRNA mediated approach of *Ddx21* transcript knockdown, we again observed a significant reduction in the number of 16-cell stage primary ICM founder cells, that was confined to the marked microinjected clone (figure 4; electronic supplementary material, figure S4b and table S4) Collectively, the data demonstrate experimental knockdown of candidate TOP-motif containing mRNAs phenocopies mTORi, and direct dysregulation of the mTOR-EIF4EBP1-EIF4E/mRNA cap-binding-complex axis, mediated impairment of primary ICM founder cell formation. They also support a hypothesis that enhanced mTORC1 signalling through the 8- to 16-cell transition potentiates translation of specific mRNA transcripts with functional roles in 16-cell stage blastomere spatial positioning.

### 2.4. mTORi impairs 16-cell stage primary but not 32-cell stage secondary ICM founder cell generation

**Figure 5.**
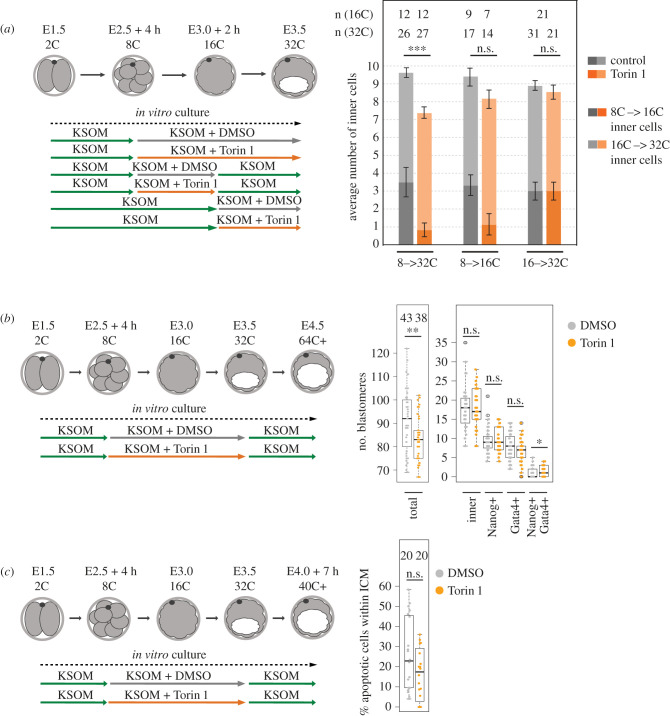
mTORi does not affect secondary ICM founder cell generation and ICM cell numbers recover during blastocyst maturation (E3.5–E4.5) after prior mTORi from the late 8-cell stage. (*a*) Experimental scheme and quantification of inner cell numbers at 32-cell stage, as contributed by primary and secondary ICM cells, +/− Torin1. Primary ICM cell count was estimated by fixation of some embryos at 16-cell stage and quantification of their number of inner cells, to allow for the number of such primary and secondary founders to be determined at the 32-cell stage, when deducting this number and allowing for the extra cell division. (*b*) Experimental scheme and quantification of total cell number, ICM cell number and the numbers of NANOG + GATA4− (EPI), NANOG− GATA4+ (PrE) and NANOG + GATA4+ ICM cells, +/− Torin1 (E4.5). (*c*) Experimental scheme and quantification of the proportion of apoptotic ICM cells (positive for cleaved Caspase-3 IF staining), +/− Torin1 (E4.0 + 7 h). In all graphs, numbers of analysed embryos are shown.

We next asked the question whether mTORC1 dependency during primary ICM founder cell generation was applicable to generation of secondary founders, after the 16- to 32-cell transition. Embryos were exposed from the pre-M-phase late-8-cell stage to mTORi (with Torin1) or solvent control (DMSO) and cultured to the mid-32-cell stage (defined morphologically as control blastocysts exhibiting cavities occupying approximately 50% of embryo volume). mTORi treated groups displayed significantly fewer inner/ICM cells than controls but to a lesser extent (i.e. as could solely be explained by the initial lack of dividing 16-cell stage primary founder ICM cells), suggesting mTORi did not impair internalization of secondary ICM founder cells (figure 5*a*; electronic supplementary material, figure S5a). Indeed, if mTORi/DMSO was administered from the mid-16-cell stage, no significant differences in ICM cell number were observed (figure 5*a*; electronic supplementary material, figure S5a), indicating a developmentally staged requirement for enhanced mTORC1 activity to appropriately segregate primary ICM founder cells that is not required during the subsequent 16- to 32-cell transition. When mTORi was limited to a period spanning the late-8-cell stage until the mid-16-cell stage and embryos further cultured under normal conditions to the mid-32-cell stage, the total number of primary inner cells (as mathematically calculated) was still impaired although the total number of inner cells was now restored to levels statistically insignificant to DMSO treated controls (although still slightly fewer), indicative of a degree of compensatory secondary ICM founder cell generation post-Torin1 removal (figure 5*a*; electronic supplementary material, figure S5a). We next asked if mTORi would affect the specification of peri-implantation stage (E4.5) blastocyst lineages, as literature reports have suggested primary ICM founders are biased to form EPI and secondary ICM founders PrE [14,36,38,42,43]. Pre-M-phase late 8-cell stage embryos were exposed to either mTORi (using Torin1) or DMSO control culture conditions until the mid-32-cell stage (concomitant with irreversible TE specification [73]) and then transferred to conventional culture media until the late blastocyst stage (E4.5); note that mTORi could not be given beyond this point as developmental diapause would result [57]. Blastocysts were then IF stained for CDX2, GATA4 and NANOG, as markers of specified TE, PrE and EPI lineages, respectively. We observed the ICM cell number was equivalent between the two groups but overall cell number in mTORi treated embryos was less, possibly indicating reduced cellular fitness in the TE cell lineage. Within the ICM, numbers of NANOG+ and GATA4− cells (indicative of EPI specification) were equivalent under mTORi versus control conditions but the number of NANOG− and GATA4+ cells (indicative of PrE differentiation) was reduced, but not significantly. Although, we also observed a significant increase in the percentage of ICM atypically co-expressing both markers (i.e. NANOG+ and GATA4+; figure 5*b*; electronic supplementary material, figure S5b), also indicative of perturbed PrE differentiation. We speculated if recovered ICM cell number in mTORi late embryos, that present with fewer ICM cells at the mid-32-cell stage, may result from reduced levels of confirmed ICM apoptosis known to occur during blastocyst maturation [38] but IF staining for cleaved Caspase-3 (at E4.0 + 7 h) did not reveal any significant decrease in mTORi treated embryos compared with DMSO controls (figure 5*c*, electronic supplementary material, figure S5c). These data indicate mTORi during the 8- to 32-cell stages, including confirmed deficits in primary ICM founders, is compensated during blastocyst maturation to ensure an appropriately sized ICM consisting of correctly specified EPI but impaired PrE differentiation. They also suggest reduced numbers of primary ICM founders do not ultimately impair EPI specification that may be inferred from other reports linking biased EPI and PrE formation to primary and secondary ICM founders, respectively [38,40] although the extent to which regulatory compensatory mechanisms, not ordinarily operative in unperturbed development, participate cannot be excluded.

### 2.5. Supernumerary outer cells in mTORi treated blastocysts exhibit ICM-like marker gene expression

**Figure 6.**
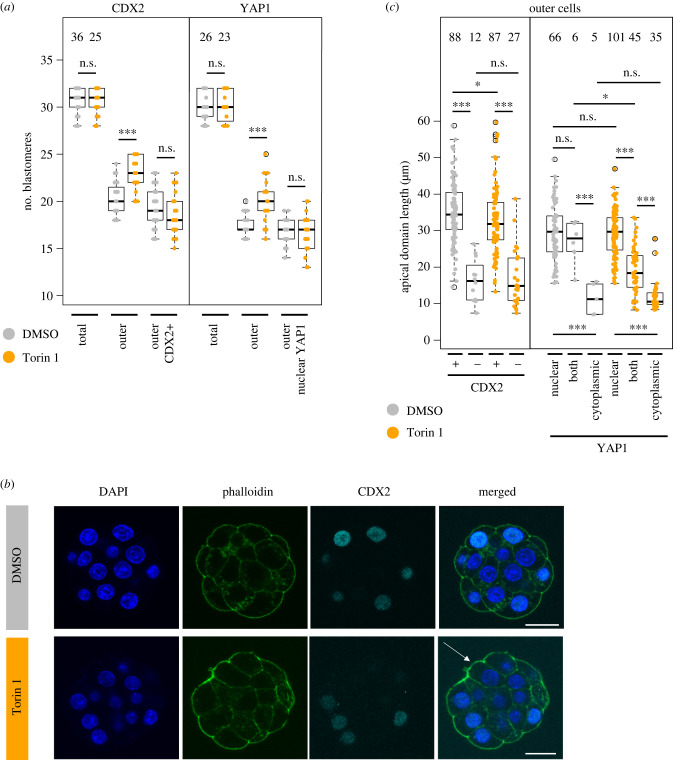
Supernumerary outer cells in mTORi 32-cell stage embryos exhibit molecular characteristics of ICM-like cells. (*a*) Quantification of total number of blastomeres, outer blastomeres, and CDX2 + outer blastomeres, or outer blastomeres with nuclear YAP1 without cytoplasmic YAP1 signal, +/− Torin1. Numbers of analysed embryos are shown. (*b*) Example IF staining micrographs of CDX2 staining in 32-cell stage embryos cultured under control and mTORi conditions. CDX2 visualized in cyan, DAPI in blue, phalloidin in green, representative confocal *z-*stacks for individual stages shown. Scale bar: 20 µm. An outer cell without CDX2 in mTORi condition marked by an arrow. (*c*) Quantification of apical domain length in single *z-*stack (with largest membrane length) in outer cells +/− CDX2 and nuclear, cytoplasmic or nuclear + cytoplasmic YAP1, +/− Torin1. Numbers of analysed blastomeres are shown.

We noted embryos exposed to mTORi (using Torin1) from the pre-M-phase late-8- to the 32-cell stage did not exhibit any significant difference in average outer cell numbers appropriately expressing CDX2 or nuclear sequestered YAP1 (indicative of suppressed Hippo-signalling), despite having supernumerary outer cells (and fewer ICM cells); figure 6*a*,*b*; electronic supplementary material, figure S6a. We speculated this might reflect differences in contactless apical domain area. We measured the length of apical domains (in maximal confocal *z*-sections) in all outer cells and found atypical CDX2− blastomeres in the mTORi treated group have significantly smaller apical domains than CDX2+ cells. Moreover, this average apical domain length was indistinguishable from that in spontaneously occurring CDX2− blastomeres in DMSO controls (figure 6*c*), albeit occurring in more outer blastomeres (a small and significant reduction in apical domain size in CDX2+ cells in the mTORi group versus the DMSO group was also observed, possibly reflecting mTORi embryos having supernumerary outer cells). We next categorized subcellular localization of YAP1 in outer cells (as either (i) exclusively nuclear, (ii) cytoplasmic and nuclear or (iii) only cytoplasmic—as a readout of Hippo-signalling activity) and compared average apical domain size in DMSO and mTORi treated embryos (figure 6*c*). No significant difference in outer blastomeres with appropriately exclusively nuclear YAP1 (associated with suppressed Hippo-signalling and TE differentiation) was seen. Neither was there any difference between the two groups in outer blastomeres with only ectopic cytoplasmic YAP1 localization. However, the average apical domain size of such cells was robustly and significantly smaller when compared with those with exclusively nuclear YAP1 within each group (again more frequently observed in the mTORi group). We term these cells medium apical domain (MAD), to distinguish them from SAD cells with only minimal contactless apical domains and interpret the data reflecting a threshold in apical domain size needed to supress Hippo-signalling. Consistently, we also observed significant, yet intermediary, reductions in apical domain size in mTORi treated embryos correlating with YAP1 localization in both the cytoplasm and nucleus (not observed in DMSO controls); again, possibly relating to such embryos having supernumerary outer cells after mTORi. These data indicate smaller apical domain sizes of MAD cells, either occurring infrequently and spontaneously in DMSO controls or with increased incidence under mTORi conditions, correlating with increased Hippo-signalling (i.e. cytoplasmic YAP1) and a lack of CDX2 expression/TE differentiation. We next assayed, in each group, extents of apical domain polarity via quantitative IF (normalized to measured apical domain area) against the polarity factor PARD6B [4], as a function of CDX2 expression. In control embryos, there were no differences in apical polarity between infrequently observed CDX2− MAD and appropriately CDX2+ outer cells. However, after mTORi we found supernumerary CDX2− outer MAD cells either exhibited apical polarity to the same extent as control group outer cells (irrespective of CDX2 status) or it was actually increased (averaging an overall significantly higher level than in CDX2+ cells of the same embryos; electronic supplementary material, figure S6b). Indeed, when PARD6 expression was measured on the whole embryo level, there were no significant differences between DMSO and mTORi treated embryos (electronic supplementary material, figure S6c). These data indicate the failure of supernumerary outer MAD cells to activate CDX2 expression (and suppress Hippo-signalling) after mTORi is not associated with defective apical polarization at the 32-cell stage and that such cells cannot appropriately specify TE in a manner germane to relative spatial position or polarity status. We hypothesized the MAD cell phenotype was caused by insufficiently large, albeit still polarized, apical domains being unable to appropriately sequester and functionally inhibit the essential embryo Hippo-pathway activator AMOT from basolateral membrane signalling domains [9,34]; thus, conferring an ectopic pseudo-inner cell phenotype, that due to the presence of apical-basolateral polarization prevents blastomere internalization. Using embryos exposed to mTORi or DMSO control from the pre-M-phase late-8- to the 32-cell stages, we assayed for ectopic localization of AMOT on outer cell lateral membranes of CDX2+ and CDX2− outer cells (note: microscopic resolution prevented objective assessment of basal AMOT localization due to proximity of inner cells with normal plasma membrane associated AMOT). However, in both groups, we did not detect enhanced lateral AMOT localization in mTORi treated embryos versus DMSO controls and neither in MAD cells (electronic supplementary material, figure S6d).

Collectively, these data indicate mTORi induced supernumerary MAD outer cells are unable to inhibit Hippo-pathway activation that would ordinarily permit nuclear accumulation of YAP1 and TE differentiation. However, while such blastomeres display smaller contactless apical domains, they nevertheless remain polarized and capable of sequestering AMOT. Therefore, the mechanism by which MAD outer cell activation of Hippo-signalling occurs and results in a pseudo-inner cell phenotype must be independent of apical-basolateral polarity and AMOT itself, potentially related to increased neighbouring MAD cell contacts. However, the fate of such MAD cells and their progeny by the late peri-implantation blastocyst stage (E4.5) remains to be resolved.

### 2.6. Conclusion

We confirm enhanced mTORC1 signalling levels around the onset of M-phase in 8-cell stage mouse embryo blastomeres, that via regulation of the mTOR-EIF4EBP1-EIF4E/mRNA cap-binding-complex axis, positively influences specific generation of primary, and not secondary, populations of ICM founder cells. Although consistent with published reports associated with regulation of apical basolateral polarity, pre-M-phase positioning of 8-cell stage nuclei and resulting mitotic spindle orientation affecting primary ICM founder generation [15,18,21,24,25], we do not observe any directly consistent and explanatory perturbations in these processes. Rather, we propose mTORi mediated mis-localization of 16-cell stage blastomeres is not directly related to such mechanisms *per se*, but manifest in impaired translation of functionally significant and specific subsets of mRNA, normally intransigent to protein translation under basal mTORC1 signalling (including, but not necessarily limited to, those containing TOP-motifs). The fact embryos partially compensate their development during blastocyst maturation (ensuring appropriate EPI specification but impaired PrE differentiation) after prior mTORi during the pre-M-phase 8- to mid-32-cell stages, is testament to their known developmental regulative capacity and maybe linked to ectopically activated Hippo-signalling in supernumerary 32-cell stage MAD cells (unrelated to the classically recognized apical-basolateral polarity model of sequestered AMOT localization [9–11,34]).

## 3. Discussion

Considerable debate exists regarding developmental history of ICM cells and their eventual fate as specified EPI or differentiating PrE (i.e. derivation of primary and secondary ICM founders). Non-invasive timelapse and lineage tracing analyses of transgenic reporter embryos have suggested primary ICM founders are biased to form EPI (in 75% of cases) and secondary ICM cells are strongly fated to yield PrE (in 85% of cases), with infrequent third-wave ICM allocation invariably fated to PrE [38]. Thus, supporting a developmental history model of ICM fate (also reviewed in [36,37]). This is contested by similar independent lineage analyses, permanently marking cell clones, revealing a lack of developmental history effect on EPI and PrE derived post-implantation tissues [39]. Subsequent comparative reanalyses of these datasets have highlighted a context dependency regarding initial numbers of primary ICM founder cells. For example, the former study reported an average of 2.8 generated primary ICM cells (contributing approx. 50% of the eventual 32-cell stage ICM), whilst the latter described formation of 4.8 such cells (resulting in 80% contribution to 32-cell stage ICM [14]; an increase possibly related to inadvertent microinjection mediated downregulation of 8-cell stage apical-basolateral polarity, known to promote cellular internalization via enhanced actomyosin contractility [16,18]). Such reappraised data raise the possibility that when 16-cell stage primary ICM founder cells are limiting, progeny are biased to form EPI but surplus contribution can spill over into PrE generation. Consistently, plots of relative percentage contributions of generated primary ICM cells to total ICM number and their eventual EPI or PrE fate support this model [14,39]. Hence, developmental history and the primary/secondary origin of ICM founder cells seem able to affect ultimate EPI versus PrE fate but is subject to context-dependent regulative parameters. This is further supported by molecular observations reporting elevated levels of *Fgf4* and *Fgfr2* transcripts in primary and secondary ICM founders, respectively [31,42]. We report elevated mTOR signalling upon 8-cell stage mitotic entry facilitates primary ICM founder generation but importantly is not required during formation of secondary ICM populations. Restriction of mTORi sensitivity to primary ICM formation (8- to 16-cell division) implies that there are distinct mechanisms underpinning generation of primary and secondary (16- to 32-cell division) ICM founder cell populations. These differences may affect their eventual fate within developing ICM. However, surprisingly when embryos were cultured under mTORi conditions from the pre-M-phase late-8- to 32-cell stages, followed by blastocyst maturation in conventional media, we found the recorded deficits in 32-cell stage ICM cell numbers (solely originating from reduced primary ICM founder contribution) were not only compensated to levels seen in control embryos (although the TE cell number was reduced) but the overall number of ICM cells correctly specified as EPI (i.e. NANOG+ and GATA4− cells) was also statistically the same, although PrE differentiation was impaired (i.e. combined reduced NANOG−/GATA4+ and enhanced NANOG+/GATA4+ cell numbers); figure 5*b*. Such data may seem counterintuitive regarding the developmental history model, as it might be expected that fewer primary ICM founders would result in fewer EPI cells. However, it may also simply reflect developmental regulatory capacity ensuring appropriate pluripotent EPI formation (as a foetal progenitor pool) at the expense of PrE differentiation, a mechanism that during unperturbed development is not normally executed. This is possibly related to lower levels of expressed FGF4, required to drive PrE differentiation [39], in the smaller ICMs of mTORi treated early blastocysts. It is therefore impossible to unequivocally conclude if 8- to 16-cell active mTOR-dependent mechanisms of facilitating primary ICM founder generation support such a developmental history related model. Although previously, we reported clonal inhibition of TE cell fate, using microinjected siRNAs specific for *Tead4* transcripts, generates excess ICM contribution favouring EPI and biased against PrE formation, respectively [43] strongly suggesting an extra approximately 12 h of polarity-dependent Hippo-pathway suppression in outer 16-cell stage blastomeres may prime derived secondary ICM founders to preferentially differentiate towards PrE. Additionally, a recent paper reports NANOG dependent coordinated expression of pluripotency-related gene expression during the 16- to 32-cell stage transition, suggesting EPI specification may actually originate (in part) in primary ICM founders [74]. Whilst not definitive, these collective data at least support an aspect of developmental history underlying ICM cell fate, that is nonetheless potentially subject to regulative and compensatory mechanisms. In this context, it is possible individual dividing 8-cell blastomere mTOR activity may act as a developmental rheostat (potentially responding to cellular metabolic status) to regulate derived primary ICM founder numbers, that can then be fine-tuned by distinct and presently unknown and/or stochastic mechanisms (e.g. involving cell–cell contacts). Thus providing embryos two opportunities to generate a germane number of ICM progenitors necessary to successfully sustain both EPI and PrE specification.

Another aspect of developmental regulation arising from mTORi from the pre-M-phase late-8- to 32-cell stages are observations of supernumerary MAD outer cells that fail to express CDX2 and exhibit atypical cytoplasmic localization of YAP1, indicative of pseudo-inner cell Hippo-pathway activation and failed TE differentiation, despite exhibiting intact apical-basolateral polarity, strongly suggesting MAD cells are undergoing regulative adaptation. As such CDX2− MAD cells exhibit statistically smaller apical domains, we considered this may result in an insufficient capacity to sequester the Hippo-pathway activator AMOT, despite their polarized status. However, we could find no evidence of ectopic lateral membrane AMOT localization that would support this model (electronic supplementary material, figure S6d). Nevertheless, the fact mTORi-induced formation of supernumerary outer MAD cells is associated with failed TE specification and activated Hippo-signalling (figure 6) indicates existence of additional mechanisms to activate Hippo-signalling independently of classically described polarity-dependent functional sequestration of AMOT to the apical domain [9–11,34]. Moreover, the fact MAD cells have reduced contactless apical domains suggests these are potentially related to mechanisms reliant on enhanced cell contact or mechanical force akin to other described contexts of Hippo-signalling regulation; e.g. regulation of tissue growth and organ formation (reviewed in [75]). Although we did observe small yet significant reductions in the basolaterally localized cell adhesion protein CDH1 in 16-cell stage embryos after mTORi (electronic supplementary material, figure S3b), it will be interesting to investigate to what extent described molecular factors underpinning these polarization independent mechanisms affect relative spatial blastomere positioning during unperturbed and regulative preimplantation mouse embryo development.

In embryos cultured under mTORi conditions from the pre-M-phase late-8- until the 32-cell stages and then cultured in conventional media to the late blastocyst (E4.5) stage, we observed impaired PrE differentiation manifested as reduced NANOG−/GATA4+ and enhanced NANOG+/GATA4+ ICM cell numbers (although the number of specified EPI cells, compared to control groups, was equivalent; figure 5*b*). The presence of GATA4+/NANOG+ cells, ordinarily not observed in control embryos, is notable and possibly resembles an uncommitted cell fate state reminiscent of NANOG and GATA6 co-expression in early blastocyst ICM [76] or an attempt to initiate PrE differentiation without downregulating pluripotency. Interestingly, we previously reported such atypical NANOG and GATA4 co-expression during mouse blastocyst maturation under p38-MAPK inhibited (p38-MAPKi) culture conditions [58–60], particularly when p38-MAPKi is supplemented by pharmacological activation of mTOR [60]. Such phenotypes are associated with impaired general protein synthesis and associated reductions in polysome formation and rRNA processing [60]. Consistently, we find if mTORi from the pre-M-phase late-8-cell stage is substituted with p38-MAPKi, we can elicit the same phenotype of fewer primary ICM founder cells (electronic supplementary material, figure S6e), further cementing an emerging importance of p38-MAPK function and its link to wider mTOR signalling in preimplantation development. However, the exact mechanistic details require further investigation.

The mTORi phenotypes of impaired primary ICM founder cell formation described here are mechanistically novel; neither being related to apical-basolateral polarity defects nor positioning of pre-M-phase 8-cell stage nuclei along the embryonic axis or altered orientation of mitotic spindles [24] (figure 3; electronic supplementary material, figure S3) and indicate functionally downstream mTORC1 mediated mechanisms regulating relative blastomere spatial positioning at the onset of the 16-cell stage. Our data denote mTORi phenotypes are, at least partially, based on temporally controlled mTORC1 regulated translation of subsets of functionally significant mRNAs, involving the mTOR substrates EIF4EBP1, LARP1 and assembly of the m^7^G-cap-binding-complex (EIF4F). Moreover, identification and functional verification of candidate TOP-motif containing transcripts encoding ANK2, DCTN2 and DDX21 (eliciting similar primary ICM founder cell deficits, after clonal RNAi-mediated knockdown; figure 4) indicates mTORC1 can regulate relative 16-cell stage blastomere positioning via potentiating specific TOP-motif containing mRNA translation (although other classes of mRNA may be affected); but exclusively at the 8- to 16-cell stage transition. Interestingly, following clonal RNAi-mediated knockdown of such TOP-motif containing mRNAs, we did not observe any enhanced compensatory contribution of the non-microinjected clone to the primary ICM population at the assayed 16-cell stage, suggesting such compensatory mechanisms associated with competition for inner spatial positions (as has been reported following experimental downregulation of apical-basolateral polarity or observed in naturally occurring apolar outer 16-cell stage blastomeres [15,18]) were yet to be invoked at the developmental point assayed. As ANK2 and DCTN2 are cytoskeletal proteins [68,70] such mechanisms likely involve cytoskeleton remodelling. Moreover, identification and validation of DDX21, an RNA-binding helicase implicated in rRNA processing and potential resolution of RNA secondary structure [71,72], is also consistent, as most characterized TOP-motif containing mRNAs encode proteins related to protein synthesis itself (reviewed in [77]). Thus, suggesting the possibility DDX21 levels contribute a positive feedback loop ensuring efficient translation of other, potentially TOP-motif containing, mRNAs. The phenocopy of mTORi mediated deficits in primary ICM cell formation using p38-MAPKi (electronic supplementary material, figure S6e) is also notable, as we previously identified DDX21 as a p38-MAPK effector protein in early mouse blastocyst development and a verified component of PrE specification [78]. Enhanced mTOR-mediated translation of *Ank2* transcripts, in proximity to condensing chromosomes and forming meiotic spindles, is reported in maturing mouse oocytes and ensures appropriate and highly asymmetric cell divisions generating the first polar body [54,55] implying potential mechanistic similarities that generate primary, but not secondary, populations of blastocyst ICM founders. The molecular signal tightly regulating functionally elevated levels of mTORC1 activity at the onset of 8-cell stage M-phase currently remains unknown. An obvious candidate is the cell-cycle dependent kinase CDK1. Increased CDK1 activity upon mouse oocyte meiotic resumption is proposed as a potential trigger for increased mTOR activity [62], possibly involving PLK1 [79]. However, functional verification of this hypothesis in cleavage stage embryos is hindered as pharmacological inhibition of either kinase results in M-phase arrest. Notwithstanding, our data collectively illustrate temporal regulation of mTORC1 activity, and the translational control of specific and functionally significant mRNA transcripts, as an emerging theme during preimplantation stage mouse embryo development. It will be of great interest to develop these findings, for example using emerging contemporary techniques optimized to directly assay single cell/embryo translatomes, reflecting mRNAs undergoing direct translation, under control and mTORi or p38-MAPKi culture conditions (e.g. using Scare sample polysome profiling [80] or Ribo-ITP [81] combined with mRNA-Seq) and in other mammalian preimplantation embryo species.

## 4. Material and methods

### 4.1. Superovulation and embryo isolation

All animal work was conducted in accordance with Act No 246/1992 Coll., on the protection of animals against cruelty under the supervision of the Central Commission for Animal Welfare, approval ID 51/2015. Derivation of all experimental embryos was conducted according to the following protocol, unless specifically stated otherwise. As previously described [43], F1 generation of eight-week female hybrid mice (generated via C57BL6 female and CBA/W strain crosses) were intraperitoneally injected with 7.5IU of PMSG (pregnant mare serum gonadotrophin; Merck) and reinjected after 48 h with 7.5IU hCG (human chorionic gonadotrophic hormone; Merck), before overnight mating with F1 or F1 hybrid transgenic mT/mG stud males (original mT/mG transgenic mouse line obtained from Jackson Laboratories, STOCK Gt(ROSA)26Sortm4(ACTB-tdTomato,-EGFP)Luo/J., https://www.jax.org/strain/007576 [82]; expressing a membrane associated Tomato fluorescent reporter, that when exposed to Cre-recombinase would be excised and replaced by a membrane GFP reporter—in the case of AMOT IF experiments). At least 4 h before dissection, culture dishes were prepared with twenty 10 µl drops of KSOM + AA medium (Embryo-Max; Millipore), covered with mineral oil (Irvine Scientific) and equilibrated in an incubator at 37°C and 5% CO_2_ atmosphere. Two-cell stage (E1.5) embryos were recovered (45–47 h post-hCG) into and washed through, on a heated stage, 20 µl drops of prewarmed (37°C) M2 media containing 4 mg ml^−1^ BSA (bovine serum albumin—Merck) and then transferred through the series of KSOM + AA culture drops of pre-equilibrated plates, before transfer into the incubator (37°C and 5% CO_2_) and *in vitro* culture to the desired developmental stage. In the case of time-lapse confocal microscopy live embryo imaging, two-cell stage embryos were similarly recovered from hybrid superovulated females (using 5IU of PMSG and 5IU of hCG; Merck), generated after crossing BDF1 male and CD1 females (each strain obtained from Anlab, Czech Republic), prior to microinjection of recombinant mRNAs (see below). Embryos were then cultured as described in KSOM + AA.

### 4.2. Embryo inhibitor treatments

In each experiment, embryos at the desired stage were divided into two equal groups (one for vehicle controls and the inhibitor treatment group). 4EGI-1 (Merck), Rapamycin (Merck) and Torin1 (Selleckchem), diluted in DMSO, were used at final KSOM + AA culture concentrations of 100 µM, 5 µM and 20 µM, respectively (with vehicle controls consisting of an equivalent volume of supplemented DMSO). Embryos were then *in vitro* cultured to the desired assay point +/− inhibitor or transferred into non-supplemented conventional KSOM + AA media for further culture, before being processed for the appropriate assay. All inhibitor/vehicle control KSOM + AA culture plates were pre-equilibrated at 37°C in a 5% CO_2_ atmosphere for at least 4 h prior to addition of embryos.

### 4.3. Two-cell stage embryo microinjection

Preimplantation mouse embryo microinjection was performed as previously described [43]. Specific predesigned Silencer-Select gene siRNAs (ThermoFisher Scientific; *Eif4g* - s101902*, Larp1* - s91534, *Ddx21* - s80158 or All-Stars negative murine control non-targeting control/NTC, from Qiagen were microinjected at a final concentration of 10 µM each). dsRNAs (targeting *Ank2*, *Dctn2* or GFP mRNAs) were in-house synthesized from T7 RNA polymerase promoter-linked PCR products, using the MEGAscript T7 *in vitro* transcription kit from ThermoFisher Scientific according to provided instructions, and were microinjected at a final concentration of 200 ng µl^−1^. Templates for dsRNAs generation were generated using the following PCR oligo primer-pairs: *Ank2* sense—taatacgactcactatagggCCTCATCGAATGCCTCACCA, anti-sense—taatacgactcactatagggTTCTCCTTGGCAGCACAGAG and *Dctn2* sense—taatacgactcactatagggGGCATTGCCAGGAATGAG, anti-sense—taatacgactcactatagggCTGTCCTCTTGGTCTTTCCAA, as designed by ERNAi design tool [83] and GFP sense taatacgactcactatagggAGAGTACAAATTTTCTGTCAGTGGAGAGG, anti-sense taatacgactcactatagggAGATGTATAGTTCATCCATGCCATGTGTA. Recombinant mRNAs were generated from T3 mediated *in vitro* transcription/IVT of cDNA inserts cloned into the vector pRN3P [84], incorporating 5′ and 3′ UTRs from the frog beta-globin gene for enhanced stability, using the ThermoFisher Scientific mMESSAGE mMACHINE T3 and poly-A-tailing kits, as instructed. mRNAs were microinjected at following concentrations: encoding HA-4Ala-EIF4EBP1 200 ng µl^−1^ (insert derived from [49]) or wild-type HA-EIF4EBP1 200 ng µl^−1^ (cloned in-house) and Histone-H2B-mCherry/YFP 50 ng µl^−1^ (cloned in-house)—as fluorescent reporter genes/confirmed microinjection markers [85]. RNAs were microinjected in either single, or both, two-cell stage embryo blastomeres (to generate control or gene-specific dysregulated clones representing 50% of embryonic cells, or the entire embryo for Q-RTPCR confirmed assessment of RNAi-mediated target gene knockdown, respectively) in suspended M2 + BSA media drops, overlaid with mineral oil using IX71 inverted-microscope (Olympus), micromanipulators (Leica) and FemtoJet microinjection system (Eppendorf). As a microinjection/clonal lineage tracer marker, all siRNA/mRNAs were co-injected with either Rhodamine-conjugated dextran beads (RDBs; final concentration 2 µg µl^−1^) or Histone H2B-RFP/YFP encoding recombinant mRNA (derived from IVT of cloned cDNAs in to pRN3P, as described above [84]) Non-microinjected embryos (1–3 per experiment) served as embryo culture sentinels for subsequent appropriate *in vitro* development in KSOM + AA (as described above). Regarding time-lapse confocal microscopy live embryo imaging experiments, recovered two-cell stage embryos were microinjected in both blastomeres, using an IM-300 Narishige microinjector on a Leica DM IL inverted microscope, with the following recombinant mRNAs encoding fluorescent reporters (prepared by IVT of pRN3P plasmid templates as described above): Histone-H2B-Venus, GAP43-CFP and alpha-Tubulin-Venus, each microinjected at 10 ng µl^−1^. Note that the correct size and integrity of IVT generated mRNA/dsRNA constructs were first confirmed on denaturing and regular agarose gels, respectively, prior to microinjection.

### 4.4. Embryo fixation and immuno-fluorescent and fluorescent phalloidin staining

Protocols were as previously described [43]; briefly, prior to fixation embryonic *zona pellucidae* were removed in prewarmed (37°C) drops of Acid Tyrode's solution (Merck) diluted in M2. Embryos were then fixed, on 1.5% agar coated culture dishes, in 20 µl drops of a 4% paraformaldehyde solution (PFA; Santa Cruz Biotechnology), overlaid with mineral oil, for 20 min at 37°C. All subsequent steps were conducted at room temperature, unless otherwise stated. In 96-well micro-titre plates, embryos were then washed through three 70 µl drops of PBST (phosphate-buffered saline with 0.15% Tween 20—Merck) and placed in 50 µl of 0.5% Triton-X100 (Merck) permeablization solution diluted in PBS for 20 min. Embryos were then washed through three 70 µl drops of PBST before being transferred to 50 µl drops of blocking 3% BSA (Merck) in PBST for 30 min. Desired primary antibody dilutions (see below) were prepared in 3% BSA PBS-T (in 5 µl volumes) solution into which embryos were transferred for overnight incubation at 4°C (overlaid with mineral oil). Following three washes through 70 µl drops of PBST, embryos were subject to a secondary 3% BSA block (1 h) and then transferred into 5 µl 3% BSA drops containing an appropriate dilution of fluorescently conjugated secondary antibody (see below), overlaid with mineral oil and incubated in the dark at 4°C for 3 h. A further three 70 µl PBS-T washing steps were repeated before embryos were DNA counter-stained using Vectashield mounting media containing DAPI (Vector). Primary antibodies (and dilutions): (a) raised in rabbit: (i) anti-phospho-EIF4EBP1 (Thr37/46—Cell Signalling Technologies no. 9459; 1:50), (ii) anti-pan-EIF4EBP1 (Cell Signalling Technologies no. 9644; 1:50), (iii) anti-phospho-EIF4EBP1 (Ser64—Cell Signalling Technologies no. 9451; 1:50), (iv) anti-phospho-EIF4EBP1 (Thr70—Cell Signalling Technologies no. 9455: 1:50), (v) anti-phospho-EIF4EBP1 (Thr37/46—Cell Signalling Technologies no. 2855: 1:200), (vi) anti-AMOT (kind gift of H. Sasaki; 1:100), (vii) anti-PRKCZ (Santa Cruz Biotechnology no. sc-216; 1:200), and (viii) anti-PARD6B (Santa Cruz Biotechnology no. sc-67393; 1:200); (b) raised in mouse: (i) anti-CDX2 (Biogenex no. MU392A-UC; 1:200) and (ii) anti-YAP1 (Santa Cruz Biotechnology no. sc-101199; 1:100). Secondary antibodies (and dilutions): (i) donkey anti-rabbit-Alexa-Fluor^647^ (Abcam no. ab150075; 1:500), (ii) donkey anti-mouse-Alexa-Fluor^647^ (Abcam no. ab150107; 1:500) and donkey anti-rabbit-Alexa-Fluor^488^ (Abcam no. ab150073; 1:500). For F-actin counter-staining, immuno-fluorescent stained embryos were subject to additional processing, before the DAPI counterstain mounting in Vectashield, as follows: following the terminal three 70 µl PBS-T washes (described above), embryos were transferred into 5 µl drops, overlaid with mineral oil, of Oregon Green^488^ Phalloidin (O7466, ThermoFisher Scientific) diluted 1 : 50 in PBST and incubated at room temperature for 30 min (in the dark). Embryos were then washed through three 70 µl drops of PBST and DNA counterstained in DAPI containing Vectashield, as described.

### 4.5. Fixed sample confocal microscopy, image analysis, cell counting and statistics

Imaging protocols were as previously described [43]. Immuno-fluorescently and/or fluorescently labelled phalloidin stained embryos, of the desired developmental stage and experimental condition, were placed in small drops of PBS on the surface of glass microscope coverslip 35 mm dishes (MatTek Corp.). Scanning confocal fluorescence imaging was conducted using an Olympus FLUOVIEW FV10i inverted confocal microscope, using experiment appropriate excitation wavelengths and emission detector settings. All embryos in comparable control and experimental groups were scanned with the same non-saturating imaging settings and exported in TIFF format for image analyses. Numbers of blastomeres were counted in FV10-ASW 4.2 Viewer software (Olympus). For 16- and 32-cell stage analyses, we first compared the overall cell numbers in different conditions and proceeded with the analysis only if there was no difference, suggesting embryos in different experimental conditions were at a developmentally equal stage. Then, the numbers of inner and SAD cells were counted only in embryos with exactly 16 cells (for 16-cell stage analyses) and 28–32 cells (for 32-cell stage analyses), to aid direct comparison. Blastomeres were divided into categories: (i) inner (the blastomere is fully inside the embryo, without contactless membrane), (ii) SAD (having minimal contactless surface, less than 5 µm contactless membrane length in the confocal *z*-stack exhibiting the maximal contactless membrane), (iii) outer (contactless membrane greater than 5 µm). A specific category of outer cells were defined as MAD cells (classified only at 32-cell stage) and comprised contactless membrane lengths between 5 and 30 µm in the confocal *z-*stack exhibiting the maximal contactless membrane. Fluorescence intensity was quantified using FIJI freeware [86], either as corrected total cell fluorescence [CTCF = integrated density – (area of selected cell × mean fluorescence of background readings)] or normalized for against the area of the quantified region in single *z*-stack, whole blastomere or embryo as specified in text. Intensity of fluorescence membrane domains was quantified as the average intensity across the domain in a single *z*-stack with the largest length of the measured domain. All quantifications were normalized for background fluorescence. Statistical analysis was performed in R 4.2.1. (https://www.R-project.org/). Data normality was first tested by Shapiro–Wilk test and values were then compared by *t*-test (normal data distribution) or Mann–Whitney test (not normal data distribution).

### 4.6. Time-lapse confocal microscopy live embryo imaging and analyses

Post-microinjected two-cell stage embryos (expressing recombinant fluorescent mRNAs, see above) were cultured to compacted 8-cell stage (E2.5 + 4 h) and transferred into KSOM + AA imaging plates (Caisson Laboratories) supplemented with Torin1 (Selleckchem, 20 µM) dissolved in DMSO (Merck, Czech Republic) or DMSO only. Complete embryo *z*-series time-lapse imaging was performed on a Leica SP5 confocal microscope, equipped with EMBL incubator set to 5% CO_2_ at 37°C. The wavelengths 458 nm, 514 nm and 561 nm were used for excitation, HCX PL APO CS 40× water objective NA 1.1 and HyD detectors were used for detection of CFP, Venus and mCherry signal, respectively. Forty-one *z*-stacks were taken every 15 min for each single embryo position. Pre-M-phase 8-cell nuclei position along radial axes and mitotic spindle angles were measured in IMARIS 6.2.1 (BitPlane). For nuclear positioning, the distance between apical domain and nucleus surface, nucleus diameter, and the distance between nucleus surface and basal domain were measured from time frame images immediately preceding nuclear envelope breakdown. Mitotic spindle angles were quantified using the coordinates of the centre of the embryo and spindle poles, immediately prior to anaphase onset.

### 4.7. O-propargyl-puromycin quantification of de novo protein synthesis

OPP staining was performed using a Click-iT Plus OPP Alexa Fluor 488 Protein Synthesis Assay Kit (ThermoFisher Scientific; cat. no. C10456). Mouse embryos were first cultured *in vitro* from E1.5 to E2.5 + 7 h in KSOM + AA as described above, and then transferred to KSOM + AA supplemented with 20 µM Torin1 dissolved in DMSO, or equivalent volume of DMSO only, and 5 µM OPP Reagent, where they were incubated for 10 min. The embryos were then immediately fixed, permeabilized, and washed as described above. Click-iT reactions were set up according to the kit manual. The fixed embryos were incubated in the reaction mixture for 25 min at room temperature in the dark. Thereafter, the embryos were asked in a 1 : 1 mixture of kit-provided wash buffer and PBST, DNA counterstained in DAPI containing Vectashield, and imaged on confocal microscope as described above (the developmental stage, either 8- or 16-cell interphase or mitosis, was noted).

### 4.8. Mass spectrometry

Employed protocols to survey the general proteome by mass spectrometry in preimplantation mouse embryos +DMSO (control) or +mTORi (+Torin1), three biological replicates each, were conducted as previously described [60]. Briefly, embryos at the desired developmental stage during 8- to 16-cell transition (E2.5 + 8 and E2.5 + 9 h) were lysed in 5 µl of SDT buffer (4% SDS, 0.1 M DTT, 0.1 M Tris/HCl, pH 7.6), incubated at 95°C for 12 min and frozen at −80°C until further processing. Individual protein solutions were processed using filter-aided sample preparation (FASP) method as described previously [60]. FASP eluates were transferred into the LC-MS vials and analysed using an Ultimate 3000 RSLCnano system connected to an Orbitrap Fusion Lumos Tribrid mass spectrometer (Thermo Fisher Scientific) as described previously [60] with the following changes. Peptides trapped on the trap column were eluted and separated on the analytical column using 130 min long nonlinear gradient programme (1–56% of mobile phase B; mobile phase A: 0.1% FA in water; mobile phase B: 0.1% FA in 80% ACN; start at 1% B, 95 min 30% B, 130 min 56% B). Mass spectrometry data were acquired in a data-dependent strategy with top 20 approach and with survey scan (350–2000 *m/z*). The resolution of the survey scan was 120 000 (at *m/z* 200) with a target value of 4 × 10^5^ ions and maximum injection time of 100 ms. HCD MS/MS (30% relative fragmentation energy) spectra were acquired with a target value of 5.0 × 10^4^. The MS/MS spectra were recorded in Orbitrap at resolving power of 15 000 (200 *m/z*) and the maximum injection time for MS/MS of 22 ms. Dynamic exclusion was enabled for 30 s after one MS/MS spectrum acquisition. The isolation window for MS/MS fragmentation was set to 1.2 *m/z*. The analysis of the mass spectrometric RAW data files was carried out using MaxQuant software (version 1.6.2.10) using default settings unless otherwise noted. MS/MS data searches were done against modified cRAP database (based on http://www.thegpm.org/crap, 112 protein sequences) containing protein contaminants like keratin, trypsin etc. and UniProtKB protein database for *Mus musculus* (https://ftp.uniprot.org/pub/databases/uniprot/current_release/knowledgebase/reference_proteomes/Eukaryota/UP000000589/UP000000589_10090.fasta.gz; downloaded 2019-05-08, version 2019/05, number of protein sequences: 22 287). Oxidation of methionine and proline, deamidation (N, Q) and acetylation (protein N-terminus) as optional modification, and trypsin/P enzyme with two allowed miss cleavages and minimal peptide length of six amino acids were set. Peptides and proteins with FDR threshold less than 0.01 and proteins having at least one unique or razor peptide were only considered. Match between runs was set for all analyses and second peptides option was checked. Protein intensities reported in proteinGroups.txt file and evidence intensities reported in evidence.txt file (output of MaxQuant program) were further processed using the software container environment (https://github.com/OmicsWorkflows), v. 3.7.1a. Processing workflow is available upon request. Briefly, it covered: (a) removal of decoy hits and contaminant protein groups, (b) protein group intensities log_2_ transformation, (c) LoessF normalization and (d) differential expression using LIMMA statistical test (qualitative changes were considered separately without statistical evaluation). Protein candidates were selected based on the following criteria: statistically significant difference (*p*-value < 0.05) in at least one of the timepoints and biological relevance based on published literature.

### 4.9. Quantitative RT-PCR

Quantitative RTPCR (Q-RTPCR) was performed essentially as described [43]. Total RNA was prepared from approximately 30 cultured 16-cell stage (E3.15) embryos that had been microinjected with gene specific or GFP/NTC control dsRNA/siRNA (as described above, i.e. in both blastomeres of late two-cell stage embryos) and total RNA purified as instructed (Arcturus Biosciences; ‘PicoPure RNA isolation’). Eluted RNA (10 µl) was DNaseI treated (Ambion; ‘DNA-free’ kit) and used to derive cDNA (30 µl) using oligo-dT priming (Invitrogen; ‘SuperscriptIII Reverse Transcriptase’). 0.5 µl of diluted cDNA (1:3—nuclease-free water) was used as template in 10 µl real-time PCR reactions (Qiagen: ‘SYBR Green PCR kit’) to assay specific transcripts (BioRad, ‘CFX96 Real-Time System’). Gene transcript specific oligonucleotide primer sequences used (final reaction conc. 400 nM): *Eif4g* (s—ACCCATGGGCAAAGCTACT, a—ACAGCATCCCCACCTTTTT), *Larp1* (s—CTCGACCCTCACCAGCAC, a—GCTCATCCTGATCCTTAGACATC), *Ank2* (s—TGAGAGTCTGCCACCTGTTG, a—TGCTCATCTTGGGGATCTTC), *Dctn2* (s—TCTGGGACCAGATGCTGCAA, a—TCAGGCCGTGAGTGGAGTTC), *Ddx21* (s—TTCCTTCTGCAACGGAAATAA, a—GAGGCACAGAATCCAAGAGC) and *H2afz* (s—GCGCAGCCATCCTGGAGTA, a—CCGATCAGCGATTTGTGGA). Transcript levels were internally normalized against *H2afz* (encoding histone H2A) levels, and fold changes (plus s.e.m.) after dsRNA/siRNA mediated knockdown derivation using the ΔΔCt method [87]. A minimum of two biological replicates of at least three technical replicates were employed.

## Data Availability

The mass spectrometry proteomics data are deposited to the ProteomeXchange Consortium via the PRIDE partner repository with the dataset identifier PXD039423. The data are provided in electronic supplementary material [88].

## References

[1] Chazaud C, Yamanaka Y. 2016 Lineage specification in the mouse preimplantation embryo. Development **143**, 1063-1074. (10.1242/dev.128314) 27048685

[2] Plusa B, Piliszek A. 2020 Common principles of early mammalian embryo self-organisation. Development **147**, dev183079. (10.1242/dev.183079) 32699138

[3] Vinot S, Le T, Maro B, Louvet-Vallée S. 2004 Two PAR6 proteins become asymmetrically localized during establishment of polarity in mouse oocytes. Curr. Biol. **14**, 520-525. (10.1016/j.cub.2004.02.061) 15043819

[4] Vinot S, Le T, Ohno S, Pawson T, Maro B, Louvet-Vallée S. 2005 Asymmetric distribution of PAR proteins in the mouse embryo begins at the 8-cell stage during compaction. Dev. Biol. **282**, 307-319. (10.1016/j.ydbio.2005.03.001) 15950600

[5] Louvet S, Aghion J, Santa-Maria A, Mangeat P. Maro B. 1996 Ezrin becomes restricted to outer cells following asymmetrical division in the preimplantation mouse embryo. Dev. Biol. **177**, 568-579. (10.1006/dbio.1996.0186) 8806832

[6] Kono K, Tamashiro DA, Alarcon VB. 2014 Inhibition of RHO-ROCK signaling enhances ICM and suppresses TE characteristics through activation of Hippo signaling in the mouse blastocyst. Dev. Biol. **394**, 142-155. (10.1016/j.ydbio.2014.06.023) 24997360 PMC4404313

[7] Mihajlovic AI, Bruce AW. 2016 Rho-associated protein kinase regulates subcellular localisation of Angiomotin and Hippo-signalling during preimplantation mouse embryo development. Reprod. Biomed. Online **33**, 381-390. (10.1016/j.rbmo.2016.06.028) 27430121

[8] Nishioka N, Yamamoto S, Kiyonari H, Sato H, Sawada A, Ota M, Nakao K, Sasaki H. 2008 Tead4 is required for specification of trophectoderm in pre-implantation mouse embryos. Mech. Dev. **125**, 270-283. (10.1016/j.mod.2007.11.002) 18083014

[9] Hirate Y et al. 2013 Polarity-dependent distribution of angiomotin localizes Hippo signaling in preimplantation embryos. Curr. Biol. **23**, 1181-1194. (10.1016/j.cub.2013.05.014) 23791731 PMC3742369

[10] Hirate Y, Hirahara S, Inoue K, Kiyonari H, Niwa H, Sasaki H. 2015 Par-aPKC-dependent and -independent mechanisms cooperatively control cell polarity, Hippo signaling, and cell positioning in 16-cell stage mouse embryos. Dev. Growth Differ. **57**, 544-556. (10.1111/dgd.12235) 26450797 PMC11520972

[11] Wicklow E, Blij S, Frum T, Hirate Y, Lang RA, Sasaki H, Ralston A. 2014 HIPPO pathway members restrict SOX2 to the inner cell mass where it promotes ICM fates in the mouse blastocyst. PLoS Genet. **10**, e1004618. (10.1371/journal.pgen.1004618) 25340657 PMC4207610

[12] Pedersen RA, Wu K, Balakier H. 1986 Origin of the inner cell mass in mouse embryos: cell lineage analysis by microinjection. Dev. Biol. **117**, 581-595. (10.1016/0012-1606(86)90327-1) 2428686

[13] Johnson MH, Ziomek CA. 1981 Induction of polarity in mouse 8-cell blastomeres: specificity, geometry, and stability. J. Cell. Biol. **91**, 303-308. (10.1083/jcb.91.1.303) 7298724 PMC2111944

[14] Morris SA. 2011 Cell fate in the early mouse embryo: sorting out the influence of developmental history on lineage choice. Reprod. Biomed. Online **22**, 521-524. (10.1016/j.rbmo.2011.02.009) 21493152

[15] Anani S, Bhat S, Honma-Yamanaka N, Krawchuk D, Yamanaka Y. 2014 Initiation of Hippo signaling is linked to polarity rather than to cell position in the pre-implantation mouse embryo. Development **141**, 2813-2824. (10.1242/dev.107276) 24948601

[16] Samarage CR, White MD, Álvarez YD, Fierro-González JC, Henon Y, Jesudason EC, Bissiere S, Fouras A, Plachta N. 2015 Cortical tension allocates the first inner cells of the mammalian embryo. Dev. Cell **34**, 435-447. (10.1016/j.devcel.2015.07.004) 26279486

[17] Watanabe T, Biggins JS, Tannan NB, Srinivas S. 2014 Limited predictive value of blastomere angle of division in trophectoderm and inner cell mass specification. Development **141**, 2279-2288. (10.1242/dev.103267) 24866117 PMC4034423

[18] Plusa B, Frankenberg S, Chalmers A, Hadjantonakis A-K, Moore CA, Papalopulu N, Papaioannou VE, Glover DM, Zernicka-Goetz M. 2005 Downregulation of Par3 and aPKC function directs cells towards the ICM in the preimplantation mouse embryo. J. Cell Sci. **118**, 505-515. (10.1242/jcs.01666) 15657073

[19] Mihajlovic AI, Bruce AW. 2017 The first cell-fate decision of mouse preimplantation embryo development: integrating cell position and polarity. Open Biol. **7**, 170210. (10.1098/rsob.170210) 29167310 PMC5717349

[20] White MD, Zenker J, Bissiere S, Plachta N. 2018 Instructions for assembling the early mammalian embryo. Dev. Cell **45**, 667-679. (10.1016/j.devcel.2018.05.013) 29920273

[21] Korotkevich E, Niwayama R, Courtois A, Friese S, Berger N, Buchholz F, Hiiragi T. 2017 The apical domain is required and sufficient for the first lineage segregation in the mouse embryo. Dev. Cell **40**, 235-247.e7. (10.1016/j.devcel.2017.01.006) 28171747 PMC5300053

[22] Dard N, Louvet-Vallee S, Maro B. 2009 Orientation of mitotic spindles during the 8- to 16-cell stage transition in mouse embryos. PLoS ONE **4**, e8171. (10.1371/journal.pone.0008171) 19997595 PMC2781390

[23] Fleming TP. 1987 A quantitative analysis of cell allocation to trophectoderm and inner cell mass in the mouse blastocyst. Dev. Biol. **119**, 520-531. (10.1016/0012-1606(87)90055-8) 3803716

[24] Ajduk A, Biswas Shivhare S, Zernicka-Goetz M. 2014 The basal position of nuclei is one pre-requisite for asymmetric cell divisions in the early mouse embryo. Dev. Biol. **392**, 133-140. (10.1016/j.ydbio.2014.05.009) 24855000 PMC4111899

[25] Humiecka M, Szpila M, Kłoś P, Maleszewski M, Szczepańska K. 2017 Mouse blastomeres acquire ability to divide asymmetrically before compaction. PLoS ONE **12**, e0175032. (10.1371/journal.pone.0175032) 28362853 PMC5376319

[26] Strumpf D, Mao C-A, Yamanaka Y, Ralston A, Chawengsaksophak K, Beck F, Rossant J. 2005 Cdx2 is required for correct cell fate specification and differentiation of trophectoderm in the mouse blastocyst. Development **132**, 2093-2102. (10.1242/dev.01801) 15788452

[27] Ralston A, Rossant J. 2008 Cdx2 acts downstream of cell polarization to cell-autonomously promote trophectoderm fate in the early mouse embryo. Dev. Biol. **313**, 614-629. (10.1016/j.ydbio.2007.10.054) 18067887

[28] Mitsui K, Tokuzawa Y, Itoh H, Segawa K, Murakami M, Takahashi K, Maruyama M, Maeda M, Yamanaka S. 2003 The homeoprotein Nanog is required for maintenance of pluripotency in mouse epiblast and ES cells. Cell **113**, 631-642. (10.1016/S0092-8674(03)00393-3) 12787504

[29] Chambers I, Colby D, Robertson M, Nichols J, Lee S, Tweedie S, Smith A. 2003 Functional expression cloning of Nanog, a pluripotency sustaining factor in embryonic stem cells. Cell **113**, 643-655. (10.1016/S0092-8674(03)00392-1) 12787505

[30] Avilion AA, Nicolis SK, Pevny LH, Perez L, Vivian N, Lovell-Badge R. 2003 Multipotent cell lineages in early mouse development depend on SOX2 function. Genes Dev. **17**, 126-140. (10.1101/gad.224503) 12514105 PMC195970

[31] Guo G, Huss M, Tong GQ, Wang C, Li Sun L, Clarke ND, Robson P. 2010 Resolution of cell fate decisions revealed by single-cell gene expression analysis from zygote to blastocyst. Dev. Cell **18**, 675-685. (10.1016/j.devcel.2010.02.012) 20412781

[32] Koutsourakis M, Langeveld A, Patient R, Beddington R, Grosveld F. 1999 The transcription factor GATA6 is essential for early extraembryonic development. Development **126**, 723-732. (10.1242/dev.126.9.723) 9895320

[33] Schrode N, Saiz N, Di Talia S, Hadjantonakis A-K. 2014 GATA6 levels modulate primitive endoderm cell fate choice and timing in the mouse blastocyst. Dev. Cell **29**, 454-467. (10.1016/j.devcel.2014.04.011) 24835466 PMC4103658

[34] Leung CY, Zernicka-Goetz M. 2013 Angiomotin prevents pluripotent lineage differentiation in mouse embryos via Hippo pathway-dependent and -independent mechanisms. Nat. Commun. **4**, 2251. (10.1038/ncomms3251) 23903990 PMC3741640

[35] Nishioka N et al. 2009 The Hippo signaling pathway components Lats and Yap pattern Tead4 activity to distinguish mouse trophectoderm from inner cell mass. Dev. Cell **16**, 398-410. (10.1016/j.devcel.2009.02.003) 19289085

[36] Zernicka-Goetz M, Morris SA, Bruce AW. 2009 Making a firm decision: multifaceted regulation of cell fate in the early mouse embryo. Nat. Rev. Genet. **10**, 467-477. (10.1038/nrg2564) 19536196

[37] Bruce AW, Zernicka-Goetz M. 2010 Developmental control of the early mammalian embryo: competition among heterogeneous cells that biases cell fate. Curr. Opin. Genet. Dev. **20**, 485-491. (10.1016/j.gde.2010.05.006) 20554442

[38] Morris SA, Teo RTY, Li H, Robson P, Glover DM, Zernicka-Goetz M. 2010 Origin and formation of the first two distinct cell types of the inner cell mass in the mouse embryo. Proc. Natl Acad. Sci. USA **107**, 6364-6369. (10.1073/pnas.0915063107) 20308546 PMC2852013

[39] Yamanaka Y, Lanner F, Rossant J. 2010 FGF signal-dependent segregation of primitive endoderm and epiblast in the mouse blastocyst. Development **137**, 715-724. (10.1242/dev.043471) 20147376

[40] Morris SA, Graham SJL, Jedrusik A, Zernicka-Goetz M. 2013 The differential response to Fgf signalling in cells internalized at different times influences lineage segregation in preimplantation mouse embryos. Open Biol. **3**, 130104. (10.1098/rsob.130104) 24258274 PMC3843820

[41] Ohnishi Y et al. 2014 Cell-to-cell expression variability followed by signal reinforcement progressively segregates early mouse lineages. Nat. Cell Biol. **16**, 27-37. (10.1038/ncb2881) 24292013 PMC4062977

[42] Krupa M, Mazur E, Szczepańska K, Filimonow K, Maleszewski M, Suwińska A. 2014 Allocation of inner cells to epiblast vs primitive endoderm in the mouse embryo is biased but not determined by the round of asymmetric divisions (8–>16- and 16–>32-cells). Dev. Biol. **385**, 136-148. (10.1016/j.ydbio.2013.09.008) 24041854

[43] Mihajlovic AI, Thamodaran V, Bruce AW. 2015 The first two cell-fate decisions of preimplantation mouse embryo development are not functionally independent. Sci. Rep. **5**, 15034. (10.1038/srep15034) 26461180 PMC4602213

[44] Fonseca BD, Smith EM, Yelle N, Alain T, Bushell M, Pause A. 2014 The ever-evolving role of mTOR in translation. Semin. Cell Dev. Biol. **36**, 102-112. (10.1016/j.semcdb.2014.09.014) 25263010

[45] Yang M, Lu Y, Piao W, Jin H. 2022 The translational regulation in mTOR pathway. Biomolecules **12**, 802. (10.3390/biom12060802) 35740927 PMC9221026

[46] Liu GY, Sabatini DM. 2020 mTOR at the nexus of nutrition, growth, ageing and disease. Nat. Rev. Mol. Cell Biol. **21**, 183-203. (10.1038/s41580-019-0199-y) 31937935 PMC7102936

[47] Saxton RA, Sabatini DM. 2017 mTOR signaling in growth, metabolism, and disease. Cell **168**, 960-976. (10.1016/j.cell.2017.02.004) 28283069 PMC5394987

[48] Beretta L, Gingras AC, Svitkin YV, Hall MN, Sonenberg N. 1996 Rapamycin blocks the phosphorylation of 4E-BP1 and inhibits cap-dependent initiation of translation. EMBO J. **15**, 658-664. (10.1002/j.1460-2075.1996.tb00398.x) 8599949 PMC449984

[49] Thoreen CC, Chantranupong L, Keys HR, Wang T, Gray NS, Sabatini DM. 2012 A unifying model for mTORC1-mediated regulation of mRNA translation. Nature **485**, 109-113. (10.1038/nature11083) 22552098 PMC3347774

[50] Yamashita R, Suzuki Y, Takeuchi N, Wakaguri H, Ueda T, Sugano S, Nakai K. 2008 Comprehensive detection of human terminal oligo-pyrimidine (TOP) genes and analysis of their characteristics. Nucleic Acids Res. **36**, 3707-3715. (10.1093/nar/gkn248) 18480124 PMC2441802

[51] Yoshihama M et al. 2002 The human ribosomal protein genes: sequencing and comparative analysis of 73 genes. Genome Res. **12**, 379-390. (10.1101/gr.214202) 11875025 PMC155282

[52] Berman AJ, Thoreen CC, Dedeic Z, Chettle J, Roux PP, Blagden SP. 2021 Controversies around the function of LARP1. RNA Biol. **18**, 207-217. (10.1080/15476286.2020.1733787) 32233986 PMC7928164

[53] Lee SE, Sun S-C, Choi H-Y, Uhm S-J, Kim N-H. 2012 mTOR is required for asymmetric division through small GTPases in mouse oocytes. Mol. Reprod. Dev. **79**, 356-366. (10.1002/mrd.22035) 22407942

[54] Susor A et al. 2015 Temporal and spatial regulation of translation in the mammalian oocyte via the mTOR-eIF4F pathway. Nat. Commun. **6**, 6078. (10.1038/ncomms7078) 25629602 PMC4317492

[55] Tetkova A, Jansova D, Susor A. 2019 Spatio-temporal expression of ANK2 promotes cytokinesis in oocytes. Sci. Rep. **9**, 13121. (10.1038/s41598-019-49483-5) 31511568 PMC6739377

[56] Li Y et al. 2021 Regulation of the mammalian maternal-to-embryonic transition by eukaryotic translation initiation factor 4E. Development **148**, dev190793. (10.1242/dev.190793) 34013332 PMC8254863

[57] Bulut-Karslioglu A, Biechele S, Jin H, Macrae TA, Hejna M, Gertsenstein M, Song JS, Ramalho-Santos M. 2016 Inhibition of mTOR induces a paused pluripotent state. Nature **540**, 119-123. (10.1038/nature20578) 27880763 PMC5143278

[58] Thamodaran V, Bruce AW. 2016 p38 (Mapk14/11) occupies a regulatory node governing entry into primitive endoderm differentiation during preimplantation mouse embryo development. Open Biol. **6**, 160190. (10.1098/rsob.160190) 27605380 PMC5043583

[59] Bora P, Thamodaran V, Šušor A, Bruce AW. 2019 p38-Mitogen activated kinases mediate a developmental regulatory response to amino acid depletion and associated oxidative stress in mouse blastocyst embryos. Front. Cell Dev. Biol. **7**, 276. (10.3389/fcell.2019.00276) 31788473 PMC6856562

[60] Bora P et al. 2021 p38-MAPK-mediated translation regulation during early blastocyst development is required for primitive endoderm differentiation in mice. Commun. Biol. **4**, 788. (10.1038/s42003-021-02290-z) 34172827 PMC8233355

[61] Bowling S et al. 2018 P53 and mTOR signalling determine fitness selection through cell competition during early mouse embryonic development. Nat. Commun. **9**, 1763. (10.1038/s41467-018-04167-y) 29720666 PMC5932021

[62] Jansova D, Koncicka M, Tetkova A, Cerna R, Malik R, Del Llano E, Kubelka M, Susor A. 2017 Regulation of 4E-BP1 activity in the mammalian oocyte. Cell Cycle **16**, 927-939. (10.1080/15384101.2017.1295178) 28272965 PMC5462087

[63] Severance AL, Latham KE. 2017 PLK1 regulates spindle association of phosphorylated eukaryotic translation initiation factor 4E-binding protein and spindle function in mouse oocytes. Am. J. Physiol. Cell Physiol. **313**, C501-C515. (10.1152/ajpcell.00075.2017) 28794108 PMC5792166

[64] Fukao A, Tomohiro T, Fujiwara T. 2021 Translation initiation regulated by RNA-binding protein in mammals: the modulation of translation initiation complex by trans-acting factors. Cells **10**, 1711. (10.3390/cells10071711) 34359885 PMC8306974

[65] Moerke NJ et al. 2007 Small-molecule inhibition of the interaction between the translation initiation factors eIF4E and eIF4G. Cell **128**, 257-267. (10.1016/j.cell.2006.11.046) 17254965

[66] Vestweber D, Gossler A, Boller K, Kemler R. 1987 Expression and distribution of cell adhesion molecule uvomorulin in mouse preimplantation embryos. Dev. Biol. **124**, 451-456. (10.1016/0012-1606(87)90498-2) 3315781

[67] Kovacovicova K, Awadova T, Mikel P, Anger M. 2016 In vitro maturation of mouse oocytes increases the level of Kif11/Eg5 on meiosis II spindles. Biol. Reprod. **95**, 18. (10.1095/biolreprod.115.133900) 27146033

[68] Stevens SR, Rasband MN. 2022 Pleiotropic ankyrins: scaffolds for ion channels and transporters. Channels (Austin) **16**, 216-229. (10.1080/19336950.2022.2120467) 36082411 PMC9467607

[69] Ayalon G, avis JQ, Scotland PB, Bennett V. 2008 An ankyrin-based mechanism for functional organization of dystrophin and dystroglycan. Cell **135**, 1189-1200. (10.1016/j.cell.2008.10.018) 19109891

[70] Urnavicius L, Zhang K, Diamant AG, Motz C, Schlager MA, Yu M, Patel NA, Robinson CV, Carter AP. 2015 The structure of the dynactin complex and its interaction with dynein. Science **347**, 1441-1446. (10.1126/science.aaa4080) 25814576 PMC4413427

[71] Henning D, So RB, Jin R, Lau LF, Valdez BC. 2003 Silencing of RNA helicase II/Gualpha inhibits mammalian ribosomal RNA production. J. Biol. Chem. **278**, 52 307-52 314. (10.1074/jbc.M310846200) 14559904

[72] Fuller-Pace FV, Nicol SM. 2012 DEAD-box RNA helicases as transcription cofactors. Methods Enzymol. **511**, 347-367. (10.1016/B978-0-12-396546-2.00016-4) 22713328

[73] Posfai E, Petropoulos S, De Barros FRO, Schell JP, Jurisica I, Sandberg R, Lanner F, Rossant J. 2017 Position- and Hippo signaling-dependent plasticity during lineage segregation in the early mouse embryo. Elife **6**, e22906. (10.7554/eLife.22906) 28226240 PMC5370188

[74] Allegre N et al. 2022 NANOG initiates epiblast fate through the coordination of pluripotency genes expression. Nat. Commun. **13**, 3550. (10.1038/s41467-022-30858-8) 35729116 PMC9213552

[75] Ma S, Meng Z, Chen R, Guan K-L. 2019 The hippo pathway: biology and pathophysiology. Annu. Rev. Biochem. **88**, 577-604. (10.1146/annurev-biochem-013118-111829) 30566373

[76] Chazaud C, Yamanaka Y, Pawson T, Rossant J. 2006 Early lineage segregation between epiblast and primitive endoderm in mouse blastocysts through the Grb2-MAPK pathway. Dev. Cell **10**, 615-624. (10.1016/j.devcel.2006.02.020) 16678776

[77] Cockman E, Anderson P, Ivanov P. 2020 TOP mRNPs: molecular mechanisms and principles of regulation. Biomolecules **10**, 969. (10.3390/biom10070969) 32605040 PMC7407576

[78] Bora P, Gahurova L, Hauserova A, Stiborova M, Collier R, Potěšil D, Zdráhal Z, Bruce AW. 2021 DDX21 is a p38-MAPK-sensitive nucleolar protein necessary for mouse preimplantation embryo development and cell-fate specification. Open Biol. **11**, 210092. (10.1098/rsob.210092) 34255976 PMC8277471

[79] Kalous J, Jansova D, Susor A. 2020 Role of cyclin-dependent kinase 1 in translational regulation in the M-phase. Cells **9**, 1568. (10.3390/cells9071568) 32605021 PMC7408968

[80] Masek T, Del Llano E, Gahurova L, Kubelka M, Susor A, Roucova K, Lin C-J, Bruce AW, Pospisek M. 2020 Identifying the translatome of mouse NEBD-stage oocytes via SSP-Profiling; a novel polysome fractionation method. Int. J. Mol. Sci. **21**, 1254. (10.3390/ijms21041254) 32070012 PMC7072993

[81] Ozadam H et al. 2023 Single-cell quantification of ribosome occupancy in early mouse development. Nature **618**, 1057-1064. (10.1038/s41586-023-06228-9) 37344592 PMC10307641

[82] Muzumdar MD, Tasic B, Miyamichi K, Li L, Luo L. 2007 A global double-fluorescent Cre reporter mouse. Genesis **45**, 593-605. (10.1002/dvg.20335) 17868096

[83] Horn T, Boutros M. 2010 E-RNAi: a web application for the multi-species design of RNAi reagents—2010 update. Nucleic Acids Res. **38**, W332-W339. (10.1093/nar/gkq317) 20444868 PMC2896145

[84] Lemaire P, Garrett N, Gurdon JB. 1995 Expression cloning of Siamois, a Xenopus homeobox gene expressed in dorsal-vegetal cells of blastulae and able to induce a complete secondary axis. Cell **81**, 85-94. (10.1016/0092-8674(95)90373-9) 7720076

[85] McGuinness BE et al. 2009 Regulation of APC/C activity in oocytes by a Bub1-dependent spindle assembly checkpoint. Curr. Biol. **19**, 369-380. (10.1016/j.cub.2009.01.064) 19249208

[86] Schindelin J et al. 2012 Fiji: an open-source platform for biological-image analysis. Nat. Methods **9**, 676-682. (10.1038/nmeth.2019) 22743772 PMC3855844

[87] Livak KJ, Schmittgen TD. 2001 Analysis of relative gene expression data using real-time quantitative PCR and the 2^−ΔΔC^_T_ method. Methods **25**, 402-408. (10.1006/meth.2001.1262) 11846609

[88] Gahurova L et al. 2023 Spatial positioning of preimplantation mouse embryo cells is regulated by mTORC1 and m7G-cap dependent translation at the 8- to 16-cell transition. Figshare. (10.6084/m9.figshare.c.6760116) PMC10409569 37553074

